# Essential Roles of BCCIP in Mouse Embryonic Development and Structural Stability of Chromosomes

**DOI:** 10.1371/journal.pgen.1002291

**Published:** 2011-09-22

**Authors:** Huimei Lu, Yi-Yuan Huang, Sonam Mehrotra, Roberto Droz-Rosario, Jingmei Liu, Mantu Bhaumik, Eileen White, Zhiyuan Shen

**Affiliations:** 1 The Cancer Institute of New Jersey, Robert Wood Johnson Medical School, University of Medicine and Dentistry of New Jersey, New Brunswick, New Jersey, United States of America; 2 Department of Radiation Oncology, Robert Wood Johnson Medical School, University of Medicine and Dentistry of New Jersey, New Brunswick, New Jersey, United States of America; 3 Department of Pediatrics, Robert Wood Johnson Medical School, University of Medicine and Dentistry of New Jersey, New Brunswick, New Jersey, United States of America; 4 Department of Molecular Biology and Biochemistry, Rutgers – The State University of New Jersey, Piscataway, New Jersey, United States of America; The Hospital for Sick Children and University of Toronto, Canada

## Abstract

BCCIP is a BRCA2- and CDKN1A(p21)-interacting protein that has been implicated in the maintenance of genomic integrity. To understand the *in vivo* functions of *BCCIP*, we generated a conditional *BCCIP* knockdown transgenic mouse model using Cre-LoxP mediated RNA interference. The BCCIP knockdown embryos displayed impaired cellular proliferation and apoptosis at day E7.5. Consistent with these results, the *in vitro* proliferation of blastocysts and mouse embryonic fibroblasts (MEFs) of BCCIP knockdown mice were impaired considerably. The BCCIP deficient mouse embryos die before E11.5 day. Deletion of the p53 gene could not rescue the embryonic lethality due to BCCIP deficiency, but partially rescues the growth delay of mouse embryonic fibroblasts *in vitro*. To further understand the cause of development and proliferation defects in BCCIP-deficient mice, MEFs were subjected to chromosome stability analysis. The BCCIP-deficient MEFs displayed significant spontaneous chromosome structural alterations associated with replication stress, including a 3.5-fold induction of chromatid breaks. Remarkably, the BCCIP-deficient MEFs had a ∼20-fold increase in sister chromatid union (SCU), yet the induction of sister chromatid exchanges (SCE) was modestly at 1.5 fold. SCU is a unique type of chromatid aberration that may give rise to chromatin bridges between daughter nuclei in anaphase. In addition, the BCCIP-deficient MEFs have reduced repair of irradiation-induced DNA damage and reductions of Rad51 protein and nuclear foci. Our data suggest a unique function of BCCIP, not only in repair of DNA damage, but also in resolving stalled replication forks and prevention of replication stress. In addition, BCCIP deficiency causes excessive spontaneous chromatin bridges via the formation of SCU, which can subsequently impair chromosome segregations in mitosis and cell division.

## Author Summary

BCCIP is a BRCA2- and p21-interacting protein. Studies with cell culture systems have suggested an essential role of *BCCIP* gene in homologous recombination and suppression of replication stress and have suggested that BCCIP defects causes mitotic errors. However, the *in vivo* function(s) of BCCIP and the mechanistic links between BCCIP's role in suppression of replication stress and mitotic errors are largely unknown. We generated transgenic mouse lines that conditionally express shRNA against the BCCIP, and we found an essential role of BCCIP in embryo development. We demonstrate that BCCIP deficiency causes the formation of a unique type of structural abnormality of chromosomes called sister chromatid union (SCU). It has been noted in the past that impaired homologous recombination and resolution of stalled replication forks can have detrimental consequences in mitosis. However, the physical evidence for this link has not been fully identified. SCU is the product of ligation between sister chromatids, likely formed as a result of unsuccessful attempt(s) to resolve stalled replication forks. Because the SCU will progress into chromatin bridges at anaphase, resulting in mitosis errors, it likely constitutes one of the physical links between S-phase replication stress and mitotic errors.

## Introduction

Loss of genomic integrity is a hallmark for tumorigenesis. Mammalian cells maintain genomic integrity by ensuring DNA replication fidelity in S-phase, equal chromosome distribution into daughter cells during mitosis, error-free repair of sporadic DNA damage throughout the cell cycle, and a coordinated cell cycle progression [1]. Homologous recombination (HR) plays roles not only in repair of DNA double strand breaks (DSB) but also in replication fidelity [2], [3]. When the replication forks stall during S-phase, one-ended DSBs are produced on one of the sister chromatids at the stalled replication fork. Subsequently, the HR machinery uses the 3′-end of a single-stranded tail of the one-ended DSB to invade the intact double-stranded DNA at the collapsed replication fork, which leads to the resolution of the stalled fork. Failure to do so causes excessive replication stress, which is often defined as the inefficient progression of the replication forks. Replication stress is a status highly susceptible to genomic instability.

The *BRCA2* tumor suppressor gene plays critical roles in HR, mainly by mediating *RAD51* function [4], [5], including the strand invasion step during the resolution of stalled replication forks. Although mutations of *BRCA2* are involved in only a small percentage of human cancers, the germline *BRCA2* mutations are of high penetrance in malignant neoplasms. This suggests that the entire molecular network of *BRCA2* is critical for cancer prevention, and defects of other proteins related to *BRCA2* may contribute to additional tumors [6]. Thus analyses of BRCA2-interacting proteins offers opportunities to identify additional genetic factors involved in tumorigenesis.

BCCIP is a BRCA2- and CDKN1A(p21)- interacting protein [7]–[10]. In human cells, two major isoforms are expressed due to RNA alternative splicing: BCCIPα and BCCIPβ [9]. Although the human BCCIPα isoform was originally identified as a p21 and BRCA2 interacting protein, later studies found that the BCCIPβ isoform also interacts with p21 and BRCA2 [10]–[12]. BCCIP down-regulation has been reported in cancers [9], [13], [14]. Human BCCIP is known to function in HR, G1/S cell cycle checkpoint, and cytokinesis [10]–[12], [15]–[18]. Furthermore, BCCIP deficiency leads to accumulation of spontaneous DNA damage and single-stranded DNA in human cells [16]. The *Ustilago maydis* homologues of BCCIP and BRCA2 (*BCP1* and *Brh2*) also interact with each other, and *BCP1* deficiency causes replication stress [19]. However, the *in vivo* function of BCCIP has not been determined.

To determine the role of BCCIP *in vivo*, we established a conditional BCCIP knockdown transgenic mouse model. We show that developmental defects in the BCCIP-deficient embryos occurred before day E6.5, and this was associated with a significant reduction of cell proliferation. In addition to an impaired repair of exogenous DNA damage, BCCIP deficiency significantly induced spontaneous chromatid aberrations that often associate with replication stress. The chromosome abnormalities in BCCIP-deficient mouse cells are characterized by the elevated formation of sister chromatid unions (SCUs) and chromatid breaks, yet a modest increase of sister chromatid exchange (SCE). This suggests an essential role of BCCIP in maintenance of chromatid stability and embryonic development in mice.

## Results

### Construction of a conditional BCCIP knockdown mouse model

**Figure 1:**
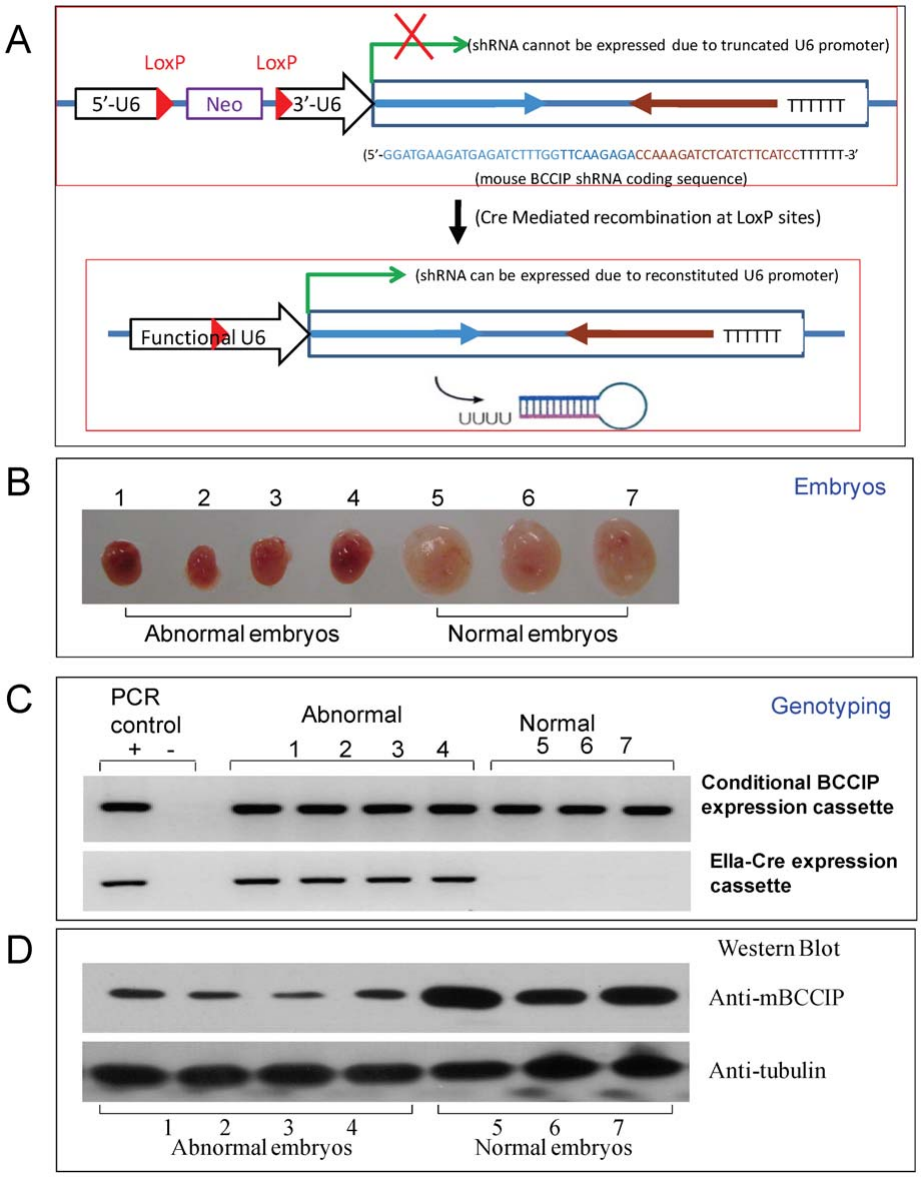
Construction of a LoxP-Cre mediated conditional BCCIP knockdown mouse line. Panel A shows the strategy for LoxP-Cre mediated conditional BCCIP knockdown in mice (see text for details). Panels B–D show the Genotyping and BCCIP expression in embryos resulted from breeding between LoxPshBCCIP^+/+^ (founder line-4) and EIIaCre^+/−^. At day E11.5, the mouse embryos were dissected, individual embryos are photographed, and shown in Panel B (number 1–4 are abnormal embryos, and number 5–7 are normal). Then, half of each of the embryos was used to extract DNA for genotyping the conditional shRNA expression and the Cre-expressing cassette (panel C). The other half was used to extract the total proteins, which were used to detect mouse BCCIP expression and β-actin as a loading control (panel D).

Although human cells express two major isoforms (BCCIPα and BCCIPβ) due to alternative RNA splicing [9], mouse tissues appear to express only the BCCIPβ isoform. In the previous studies, human BCCIP has been shown to function in DNA repair, cell cycle regulation, cytokinesis, and maintenance of chromosome stability [7], [10]–[12], [15]–[18]. Reduced or absence of BCCIP expression have been reported in human cancers [9], [13], [14], [20]. To further understand BCCIP's role in development and tumorigenesis, we generated a mouse model with BCCIP deficiency. Similar to the human *BCCIP* gene structure [9], the mouse uroporphyrinogen III synthase (*UROS*) is “head-to-head” with the *BCCIP* gene, and the *UROS* promoter is located in the intron of the *BCCIP* gene. The mouse DEAD/H box polypeptide-32 (*DDX32*) gene is “tail-to-tail” with the *BCCIP* gene. We adapted the RNAi based conditional knockdown approach developed by Coumoul and colleagues [21]–[23]. Briefly, the U6 promoter that normally drives the expression of short hairpin RNAs (shRNAs) is disrupted by insertion of a LoxPneoLoxP cassette, thus is only functional upon the conditional deletion of the LoxPneoLoxP cassette (Figure 1A). The conditional shRNA expression construct against *BCCIP* gene was integrated into the mouse genome using standard transgenic mouse techniques. Two founder homozygous transgenic mouse lines with the conditional expression cassette were generated. The two independent homozygous transgenic lines, designated *LoxPshBCCIP^+/+^-4* and *LoxPshBCCIP^+/+^-13*, were fertile, grow normally, and have the same lifespan as wild type mice. The *LoxPshBCCIP^+/+^* transgenic mice were crossed with a mouse line expressing Cre recombinase to “pop-out” the LoxPneoLoxP segment. As reported elsewhere [23], the single LoxP site left in the U6 promoter after Cre-recombination does not affect the U6 promoter activity. This reconstitutes the U6 promoter activity, leading to the expression of the anti-BCCIP shRNA (Figure 1A), to achieve a Cre-dependent conditional knockdown of BCCIP.

To verify the BCCIP knockdown, MEFs from the two founder lines were established. As predicted and shown in Figure S1, mouse cells only express one isoform. Expression of Cre in the MEFs derived from both mouse founder lines cells efficiently knocked down BCCIP (Figure S1). These MEF cells, designated *MEF4-LoxPshBCCIP* and *MEF13-LoxPshBCCIP*, were used further *in vitro* studies. It should be pointed out that BCCIP can be knocked down in heterozygous *LoxPshBCCIP^+/−^* cells by expression of Cre because one copy of the LoxPshRNA cassette is able to express shRNA against BCCIP.

### EIIa-Cre mediated knockdown of BCCIP causes embryonic lethality

In an attempt to generate mice with BCCIP knockdown, we bred the FVB/N *LoxPshBCCIP^+/+^-4*, and *LoxPshBCCIP^+/+^-13* with the FVB/N *EIIaCre^+/−^* mouse [24] that carries a Cre transgene under the control of the adenovirus EIIa promoter. The EIIa promoter drives the expression of Cre recombinase early in embryogenesis [24]. As shown in Table 1, breeding between wild type with EIIaCre^+/−^ mice resulted in approximately 1∶1 ratio of *LoxPshBCCIP^−/−^*;*EIIaCre^+/−^* and *LoxPshBCCIP^−/−^*;*EIIaCre^−/−^* mice. However, breeding of *LoxPshBCCIP* ^+/+^ with EIIaCre^+/−^ mice resulted in a significantly smaller number of *LoxPshBCCIP^+/−^*;*EIIaCre^+/−^* than *LoxPshBCCIP^+/−^*;EIIaCre^−/−^ newborns. In addition, the litter size (5.1 for founder line-4 or 6.6 for founder line-13) from the breeding between *LoxPshBCCIP* ^+/+^ and *EIIaCre^+/−^* was significantly smaller than that of wild type (*LoxPshBCCIP* ^−/−^) mice (10.3/litter). These data suggest that down-regulation of *BCCIP* causes embryonic lethality. Although there were significantly less Cre positive mice with this breeding scheme, it was noted that some Cre positive mice were viable. However, further analyses confirmed that some of these viable *LoxPshBCCIP^+/−^*;*EIIaCre^+/−^* newborn mice had lost the *LoxBCCIPshRNA* cassette (data not shown). This suggests that the *LoxBCCIPshRNA* cassette in mice is subject to spontaneous loss.

**Table 1:** Genotype distribution of newborns from cross-breeding between the two conditional LoxPshBCCIP^+/+^ mouse lines with the EIIaCre^+/−^ mice.

	No. (%) of (*EIIaCre^−/−^*)newborns	No.(%) of (*EIIaCre^+/−^*)newborns	Total No. of newborns	Average litter size
Wild type (7 litters)	35 (49%) (*LoxPshBCCIP^−/−^*)	37 (51%) (*LoxPshBCCIP^−/−^*)	72 (100%)	10.3
*LoxPshBCCIP^+/+^-4* (15 litters)	68 (89%) (*LoxPshBCCIP^+/−^*)	8 (11%) (*LoxPshBCCIP^+/−^*)	76 (100%)	5.1
*LoxPshBCCIP^+/+^-13* (14 litters)	68 (74%) (*LoxPshBCCIP^+/−^*)	24 (26%) (*LoxPshBCCIP^+/−^*)	92 (100%)	6.6

To confirm that BCCIP knockdown causes embryonic lethality, embryos from crosses between *LoxPshBCCIP* ^+/+^ *-4* and EIIaCre^+/−^ were analyzed at day E11.5. As exemplified by Figure 1B–1D, among a total of seven embryos of the same litter, four (labeled as No. 1–4 in Figure 1B) were abnormal and three (labeled as No. 5–7 in Figure 1B) were normal. The abnormal embryos have the EIIaCre-positive genotype, while the normal embryos are EIIaCre-negative (Figure 1C). As expected, the expression of BCCIP in the abnormal embryos was clearly down-regulated, while the healthy embryos expressed normal levels of BCCIP protein (Figure 1D). Analysis of Cre-dependent conditional knockdown embryos derived from another founder line (*LoxPshBCCIP* ^+/+^ *-13*) is shown in Figure S2. Altogether, these data suggest that down-regulation of BCCIP during embryogenesis causes embryonic lethality prior to day E11.5. Figure 1, Figure S1, and Figure S2 also illustrate that our conditional knockdown strategy indeed achieved the anticipated down-regulation of BCCIP upon expression of Cre-recombinase in the conditional transgenic mice.

### Development retardation starts at approximately day E6.5 in BCCIP-deficient embryos

**Figure 2:**
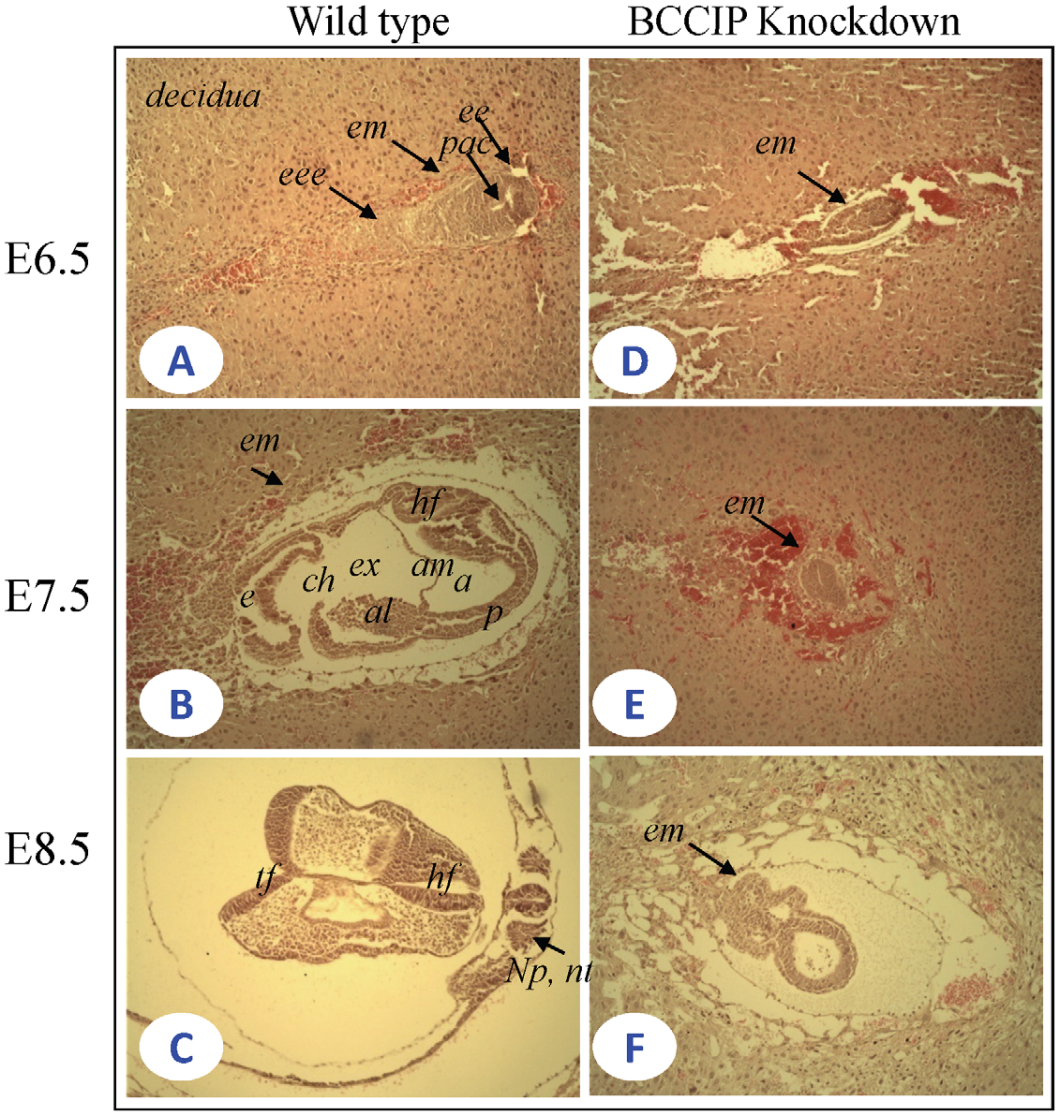
Histological sections of wild-type and BCCIP knockdown embryos. The uteri of female were dissected between 6.5–8.5 days after homologous *LoxBCCIPshRNA* ^+/+^-4 mice were mated with EIIaCre (+/−) heterozygous mice. All uterine decidual were sectioned transversely. Serial 5 µm sections were prepared. Shown are image of HE stains at with 10×10 fold magnification. A–C: wild-type embryos; D–F: BCCIP knockdown embryos. A and D: E6.5 embryos; B and E: E7.5 embryos; C and F E8.5 embryos. *eee*: extra embryonic ectoderm; *pac*: proamnotic cavity; *al*: allantois; am: amnion; *ch*: chorion; *ee*: embryonic ectoderm; *em*: embryo mass; *e*: ectoplacental cavity; *a*: amnonic cavity; *ex*: exocoelom; *p*: primitive streak; *hf*: head fold; *tf*: tail fold; *np*: neural plate; *nt*: notochord. The rest of the tissues are decidual tissue.

Given our observations that BCCIP down-regulation causes developmental arrest at day E11.5, we anticipate the anomaly in embryonic development initiates a few days prior. To define the precise timeframe for the effects of BCCIP knockdown in early embryogenesis, we analyzed embryos at different timepoints, including embryonic days E6.5, E7.5, and E8.5. As shown in Figure 2A–2C, wild type embryos were well developed during this period. By E6.5, wild-type embryos (Figure 2A) displayed normal growth and egg cylinder elongation, extraembryonic and embryonic ectoderm and pro-amniotic cavities. By day E7.5 (Figure 2B), wild-type embryos underwent gastrulation; the amniotic cavity was sealed off and three distinct cavities (amniotic cavity, exocoelom, and ectoplacetal cleft) were well developed. The neural plate, a developed notochord, a confined head and tail folds were visible at day E8.5 in a wild type embryo (Figure 2C). The mid-trunk region remained apparently attached to yolk sac, which is consistent with normal mouse embryo development [25], [26]. However, in the BCCIP knockdown embryos, there was a significantly delayed and abnormally developed embryos as evidenced by the mass size of the embryonic tissues at day E6.5 (Figure 2D). At day E7.5 and E8.5 (Figure 2E and 2F), the BCCIP knockdown embryos were developmentally retarded. There was no apparent formation of amniotic cavity, and no mesoderm differentiation at day E7.5 (Figure 2E). Also, development of the neural plate and notochord was not evident at day E8.5 (Figure 2F). These morphological observations suggest that the developmental defects caused by BCCIP knockdown in the analyzed mouse embryos are likely initiated before day E6.5.

During mouse embryogenesis, mesoderm development occurs around day E6.5. Brachyury can be used as a marker of the primitive streak, nascent mesoderm, the node and notochord [27]–[29]. To confirm that the developmental delay occurs prior to day E6.5, we examined the expression of the Brachyury protein by immunohistochemistry (IHC) at day ∼E6.5. As shown in Figure S3A, the Brachyury expression was readily detectable in the primitive streak and mesoderm in wild-type embryos, which is a sign of mesoderm differentiation (Figure S3A). However, in BCCIP deficient embryos of the same age, little Brachyury expression was detected (Figure S3B). This confirms that the embryonic development retardation in BCCIP deficient mice was likely initiated prior to day E6.5.

### BCCIP deficiency impairs embryonic cell proliferation

**Figure 3:**
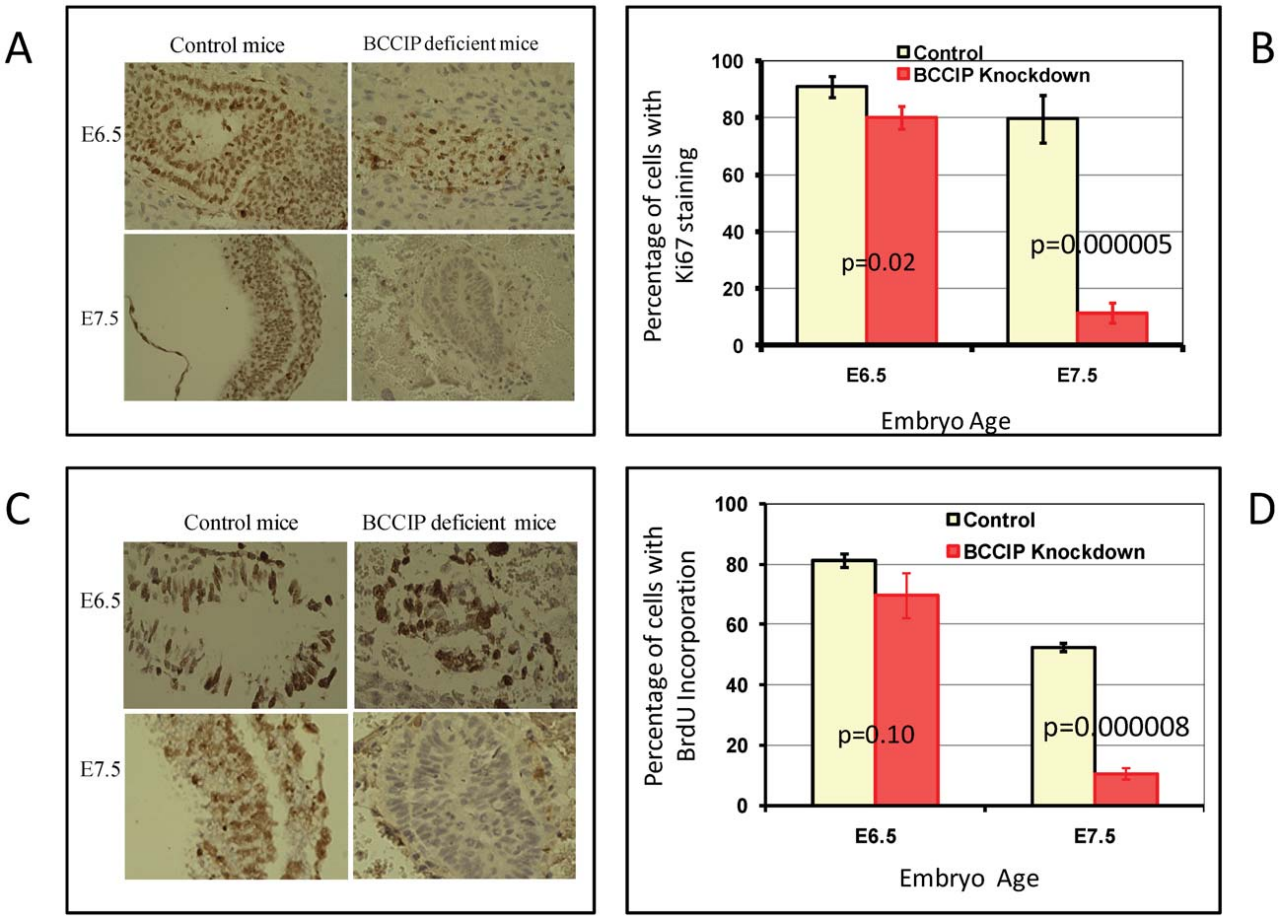
Proliferation defects in BCCIP deficient embryos. At E6.5 and E7.5, embryo tissue sections were prepared to stain for cell proliferation markers. In panels A and B (Ki67 staining): embryo sections were immuno-stained with anti-Ki67 antibody. Panel A shows a set of representative staining of Ki67 for control and BCCIP deficient (indicated on the top) mice at the ages E6.5 and E7.5 (indicated on the left). Panel B shows the percentage of embryo cells with positive Ki67 staining, and error bars represent the standard deviation of 3–5 individual counting of slides. In panel C and D (BrdU incorporation): one hour after the pregnant mice were injected with BrdU, the embryo tissues were processed to stain for BrdU (see Materials and Methods for details). Panel C shows a set of representative staining of the embryo tissue for control and BCCIP deficient (indicted on the top of the panel) at days E6.5 and E7.5 (indicated on the left of the panel). Brown nuclei are BrdU positive staining, blue nuclei are negative staining. Panel D shows the percentage of embryo cells with positive BrdU incorporation and error bars represent the standard deviation of 3–5 individual counting of slides.

As shown in Figure 2 and Figure S3, the BCCIP knockdown embryos display histological development defects around day ∼E6.5. Ki67 expression is commonly regarded as a proliferation marker. To determine whether cellular proliferation is impaired in BCCIP deficient embryos at about the same time, Ki67 expression in embryonic tissues was assessed by IHC (Figure 3A). A proliferative index, defined as the ratio of the number of Ki67-positive nuclei in the embryo tissue preparations over the total nuclei number, was determined (Figure 3B). As shown in Figure 3A and 3B, there was only a slight reduction of Ki67 expression in BCCIP knockdown embryos when compared to wild type embryos at day E6.5. However at day E7.5, the proliferation index was significantly reduced, from ∼80% in wild type to ∼11% in the BCCIP knockdown embryos (Figure 3B).

To confirm the cell proliferation assessment data, incorporation of 5-bromo-2′-deoxyuridine (BrdU) into DNA during the S phase of the cell cycle was measured at days E6.5 and E7.5. As shown in Figure 3C and 3D, there was little difference in labeling index at day E6.5 between wild type and BCCIP knockdown embryos. However at day E7.5, wild type embryos had 52% BrdU-positive nuclear staining compared to 10% in BCCIP knockdown embryos (Figure 3D). These results strongly suggest that the proliferation defects of BCCIP deficient embryos are initiated by day E6.5, consistent with the data from histological analyses (Figure 2 and Figure S3).

### BCCIP knockdown causes apoptosis around day E7.5

**Figure 4:**
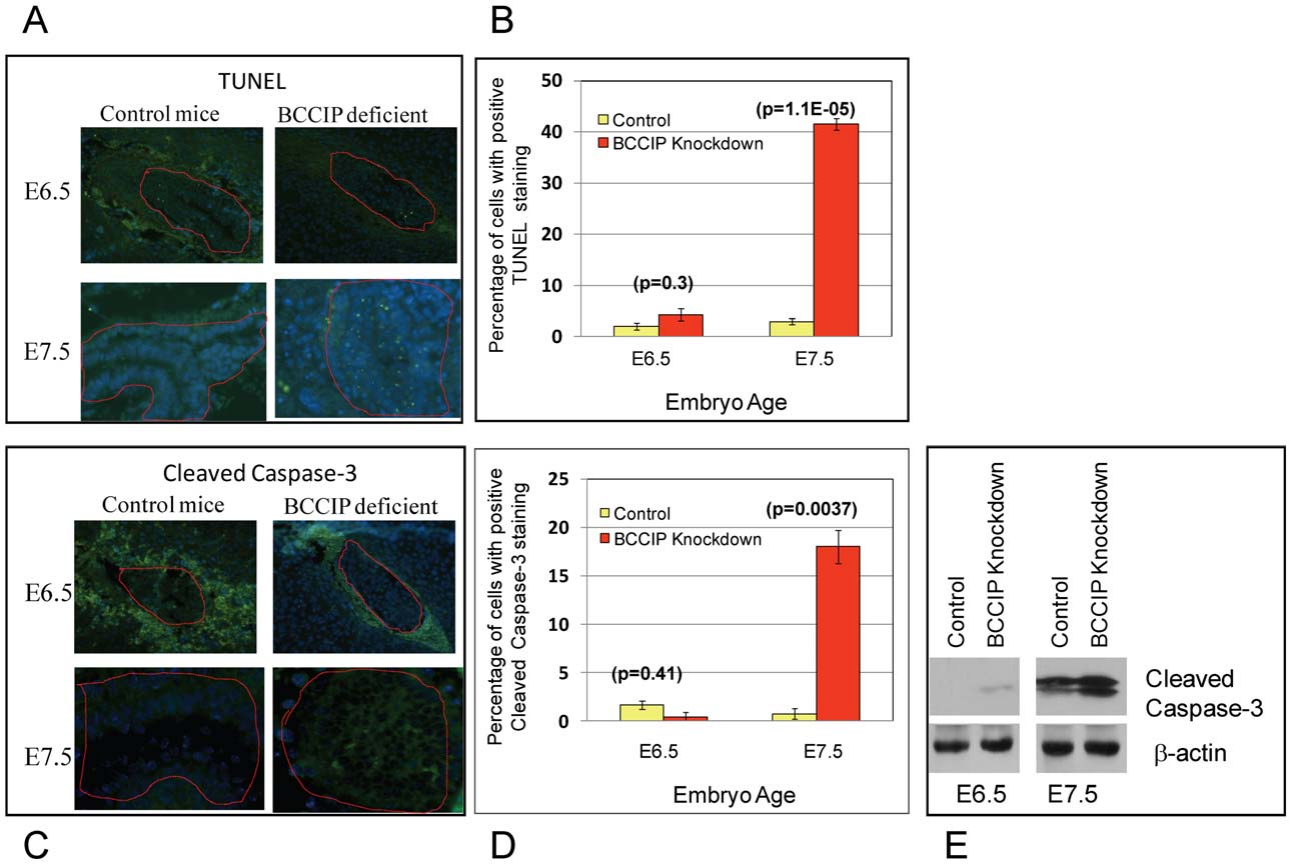
Apoptosis in BCCIP deficient embryos. At days E6.5 and E7.5, embryo tissue sections were prepared to stain for apoptosis markers. TUNEL (panel A) and anti-cleaved caspase-3 (panel C) staining were performed on embryonic tissue sections at days E6.5 and E7.5 (indicated on the left of the panels). At this stage, the surrounding the embryos are the decidual tissues that normally undergo apoptosis, and the embryonic tissues are marked with red lines. The percentage of apoptotic cells are shown in panel B and D. Panel E is a Western blot showing the increase of cleaved caspase-3 in the BCCIP deficient embryos.

To determine if the growth defect of BCCIP deficient embryos is associated with an excessive level of programmed cell death, embryo serial tissue sections at days E6.5 and E7.5 were analyzed by terminal deoxynucleotidyl transferase (TdT)-mediated dUTP nick end labeling assay (TUNEL) and anti-cleaved caspase-3 staining. At day E6.5, there was little apoptotic and caspase-3-positive cells in the wild type and the BCCIP deficient embryos (Figure 4). However, at day E7.5, clear apoptotic signals were detected in BCCIP deficient but not in wild type embryos (Figure 4). This indicates that programmed cell death in BCCIP knockdown embryos is increased as early as day E7.5, which is in strong agreement with the impaired embryo development around this time as shown in Figure 2.

### BCCIP knockdown causes blastocyst growth retardation in vitro

**Figure 5:**
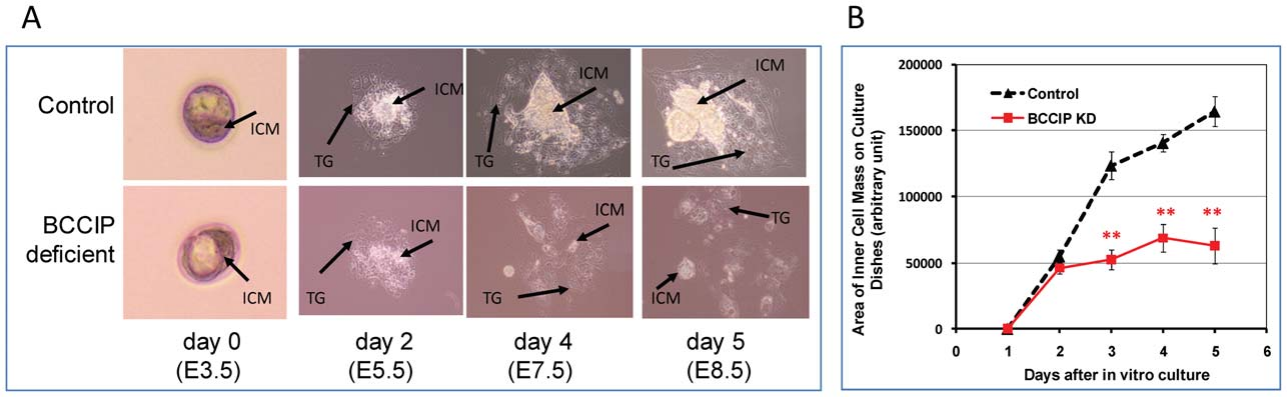
Proliferation defects of BCCIP-deficient blastocysts. Panels A and B: wild-type (control) blastocysts and BCCIP deficient blastocysts were cultured *in vitro*. Panel A shows representative images of the cultured wild-type (control, top row) and BCCIP deficient blastocysts (bottom row) at days 0, 2, 4 and 5 *in vitro* (equivalent to E3.5, E5.5, E7.5 and E8.5 *in vivo*). *ICM*: inner cell mass; *TG*: trophoblast. Panel B illustrates the average area of inner cell mass (ICM) formed in culture by the control and BCCIP deficient blastocysts. Shown are the averages of 28 blastocysts from each group, and error bars indicate the standard errors of the averages. There is no significant difference on day 2 (p = 0.18), but all p-values are less than 1.0E-06 for days 3, 4, and 5 in culture.

In early mouse embryogenesis, prior to the implantation, the inner cell mass (ICM) inside the blastocysts forms one of the earliest structures of embryos, and eventually give rise to the definitive structures of the embryo. *In vitro* Blastocyst outgrowth offers an opportunity to observe ICM growth and to assess the early post-implantational development. To assess the role of BCCIP in embryonic development prior to day E6.5, *LoxPshBCCIP^+/+^* mice were bred with EIIaCre^+/+^ mice (breeding between *LoxPshBCCIP^+/+^* and with EIIaCre^−/−^ as the control). Blastocysts were collected by uterine flushing at day E3.5, and cultured *in vitro*. The growth of ICM from the blastocysts was monitored daily while in culture. The numbers of blastocysts analyzed are summarized in Table S1. Among 71 BCCIP knockdown blastocysts, 28 (or 39%) successfully attached to the culture dish, which was a slightly lower frequency than the control blastocysts (28/58, or 48%). For the attached blastocysts, there was little morphological difference between control and BCCIP deficient blastocysts after one day in culture (equivalent to day E4.5 *in vivo*). Figure 5A illustrates the representative growth morphology of the blastocysts in culture at days 2, 4, and 5. Normally, the blastocysts hatch from the zona pellucida around day 1 to 2 in culture. As shown in Figure 5A, there was little apparent morphological difference at day 2 shortly after blastocysts hatching *in vitro*. After day 2 in culture, growth of the ICM from the BCCIP deficient blastocysts was clearly defective, although the difference in trophoblast giant cell growth between control and BCCIP deficient cells appears to be less significant (Figure 5A). To quantify the growth of the ICM *in vitro*, the relative areas of ICM were calculated using the ImageJ program. As shown in Figure 5B, the growth of BCCIP deficient ICM was significantly impaired when compared with wild type blastocysts starting at day 3 (equivalent to day 6.5 *in vivo*) in culture. These results imply that BCCIP defects affect the ICM growth, which is consistent with the *in vivo* observation of growth retardation in BCCIP knockdown embryos as described in Figure 2.

### Down regulation of BCCIP impairs growth of the MEFs

**Figure 6:**
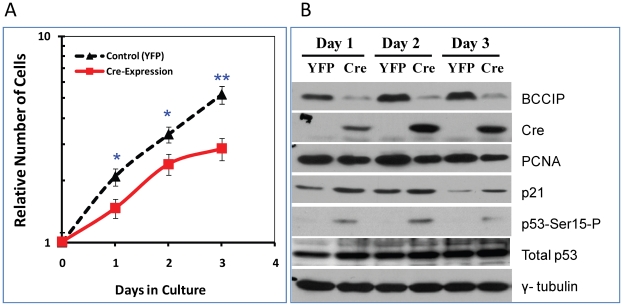
Characterization of BCCIP-deficient mouse embryo fibroblasts (MEF). The MEF4-LoxPshBCCIP (passage 1) cells with the conditional BCCIP knockdown cassette were infected with control retro-viruses that express YFP, and the Cre-expressing retrovirus to activate the knockdown of BCCIP. Then, the cells were selected by puromycine, re-plated, and the numbers of cells were counted each day thereafter. Panel A shows the relative number of cells after Cre-expression. Panel B shows the expression of BCCIP, Cre-recombinase, total p53, Ser-15 phosphorylated p53 (p53-Ser15-P), p21, and PCNA in the MEF cells at different time after the virus infection. The p-value of t-test are indicated as (*): p<0.05 and (**): p<0.01.

The *in vivo* and *in vitro* data above have shown growth retardation in BCCIP knockdown embryos, suggesting that the mouse BCCIP is essential for cell proliferation and growth. To investigate the underlying mechanism(s), we used the *MEF4-LoxPshBCCIP* cells. At passage 1, the MEF4-LoxPshBCCIP cells were infected with retroviruses expressing Cre-recombinase to reconstitute the functional U6 promoter in order to achieve BCCIP knockdown. The control groups were infected with retrovirus expressing the YFP. As shown in Figure 6A, the MEF4-LoxPshBCCIP cells infected with Cre-virus grew slower than those infected with YFP expressing virus (Control). This slowed growth of BCCIP knockdown MEF cells is coincident with a reduced level of PCNA (a proliferation marker) and increased level of p21 (Figure 6B). We also observed an increase of Ser-15-phosphorylated p53 in the BCCIP knockdown MEFs (Figure 6B), suggesting a spontaneous activation of DNA damage signaling in the BCCIP deficient cells.

### BCCIP-deficient MEFs are sensitive to radiation damage and replication stress

**Figure 7:**
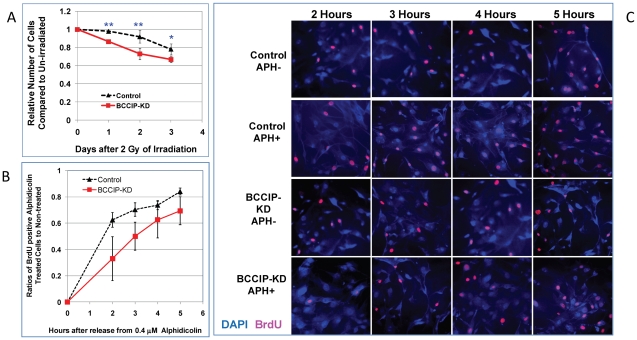
BCCIP-deficient MEF cells are more sensitive to irradiation and replication stress. The primary MEF culture from the conditional BCCIP knockdown mice were infected with virus expressing Cre-recombination (BCCIP-KD). Control was infected with viruses expressing YFP. Two days after the infection, the cells were selected with puromycin for 2 days to remove the un-infected cells. The infected cells were re-plated for radiation sensitivity and replication stress testing. Panel A shows the relative numbers of viable cells at different days after irradiation with 2 Gy of Cs-137 γ–rays, normalized to the same cells without irradiation. Panel B shows the ratio of BrdU incorporated cells between treated and non-treated cells after 30 hours of 0.4 µM alphidicolin treatment. Panel C are representative images of DAPI (blue color) and BrdU labeled cells (pink color) at different hours after release from alphidicolin treatment. The p-value of t-test are indicated as (*): p<0.05 and (**): p<0.01.

We further assessed the roles of mouse BCCIP in DNA damage sensitivity. Because of poor colony formation by the primary MEF culture, a clonogenic survival assay was technically infeasible. Thus, we performed growth inhibition assay to assess the MEF's response to modest dose of irradiation. As shown in Figure 7A, BCCIP knockdown cells exhibited greater growth inhibition by irradiation compared to control MEFs. Irradiation with 1–4 Gy of γ-rays showed a similar trend of inhibition of cell growth (Figure S4).

Under physiological conditions, without exogenous DNA damage, HR is thought to play a major role in relieving replication stress. We treated the MEF cells with low concentrations of alphidicolin (APH). After washing off the APH, cells were immediately incubated with Bromodeoxyuridine (BrdU) in APH-free medium. At various time points, the fraction of cells with BrdU incorporation was scored after immunofluorescent staining (see Materials and Methods), which reflects the recovery from replication blockage. As show in Figure 7B, when normalized to the un-treated cells, the re-incorporation of BrdU was less efficient among BCCIP knockdown cells than the control MEFs, reflecting a delayed recovery from replication stress. Figure 7C shows representative fields of BrdU-labeled cells. Together, these data suggest that the BCCIP deficient cells are not only more sensitive to DNA damage but also less efficient to recover from replication stress than control cells.

### Impaired DSB repair in BCCIP-deficient MEFs

**Figure 8:**
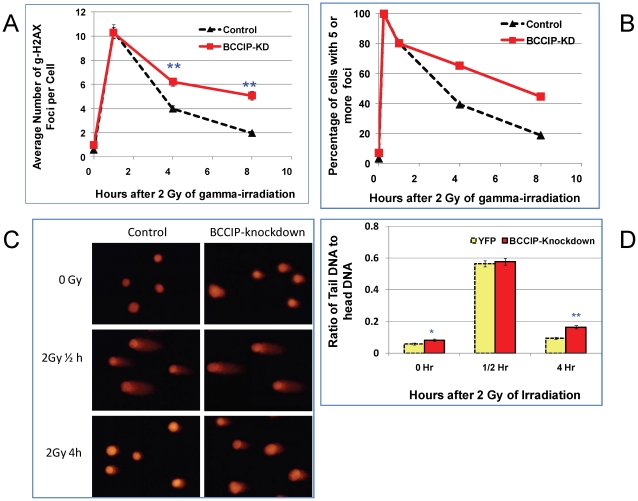
BCCIP-deficient MEF cells have impaired repair of DNA damage induced by ionizing radiation. MEF cells infected with control (YFP) and Cre-expressing viruses were irradiated with 2 Gy of Cs-137 γ-rays. At various time after the irradiation, the cells were stained for gH2AX foci. The single cell gel electrophoresis alkaline Comet assay was used to assess the residual level of DNA damage at 0.5 h and 4 h after 2 Gy of irradiation. The comet images were captured by fluorescence microscope and quantified for at least 100 cells per slide with the Cometscore software to calculate the ratio of DNA in the tails with that of the heads, which represent the relative level of residual damaged DNA. Panel A shows the average number of γH2AX foci per cells based on scoring 100–300 individual cells per sample. The p-value for each time points are: 0.17 (0 hr), 0.20 (1/4 hr), 0.85 (1 hr), 2.1E-07 (4 hr), and 2.8E-14 (8 hr). Panel B shows the percentage of cells with 5 or more γH2AX foci. Panel C shows representative comet images at different time after 2 Gy of radiation. Panel D shows the average ratio of DNA content in between the tails and the bodies based on analyses of individual cells in each group. The p-values for each time points are: 0.024 (0 hr, representing spontaneous DNA damage), 0.69 (1/2 hour), and 1.5E-08 (4 hr). As shown here, shortly after the irradiation, the amounts of damaged DNA (ratio of tails to heads) were similar. After 4 hours of repair, there is more residual damaged DNA in the BCCIP knockdown cells than the control.

To directly assess the DSB repair capability, we measured the kinetics of γH2AX removal following irradiation. As shown in Figure 8A, 15 min after irradiation, all cells have a similar level of γH2AX. However, at 4 and 8 hours after irradiation, the BCCIP knockdown MEFs have significantly more γH2AX nuclear foci than the control MEF cells. Similarly, the fractions of cells with 5 or more γH2AX foci were higher in the BCCIP knockdown cells than the controls (Figure 8B). Figure S5 shows representative γH2AX foci at different times after irradiation. These observations indicate that the control cells remove DSBs more efficiently than BCCIP knockdown MEFs, and that down regulation of BCCIP impairs DSB repair after irradiation. In addition, an alkaline comet assay revealed more residual DNA damage at 4 hours after irradiation in the BCCIP deficient MEFs when compared to control cells (Figure 8C and 8D). These data strongly suggest an impaired repair capability in the BCCIP deficient cells, consistent with the slower growth of the BCCIP knockdown MEFs after irradiation (Figure 7A).

**Figure 9:**
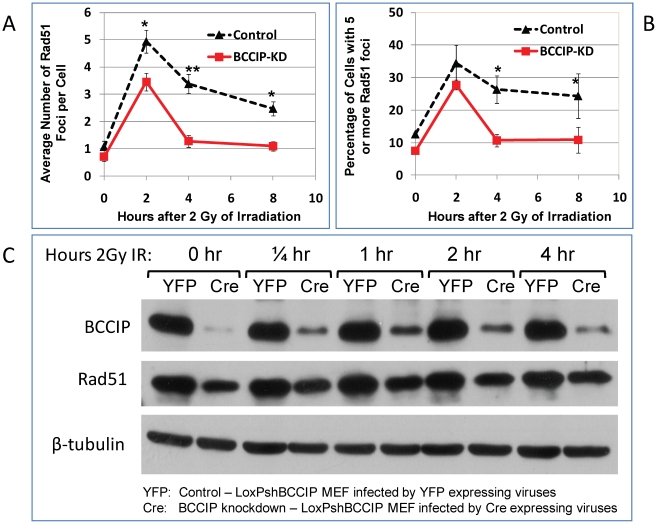
Reduced Rad51 protein level and focus formation in BCCIP-deficient MEFs. At 2, 4, and 8 hours after 2 Gy of irradiation, the mouse Rad51 was stained and the numbers of Rad51 nuclear foci were scored. Panel A shows the average number of Rad51 foci per cell at different time after 2 Gy of irradiation. Panel B shows the percentage of cells with 5 or more Rad51 foci. Panel C is a Western blot illustrating reduced Rad51 protein level of whole cell extract in the non-irradiated (0 hr) and irradiated cells at different times after irradiation.

Because human RAD51 focus formation is associated with BCCIP [10], [30], we further assessed the potential role of BCCIP in mouse Rad51 response to radiation. As shown in Figure 9A, BCCIP deficiency resulted in a significant reduction of Rad51 foci in response to radiation. Furthermore, there was a reduction of Rad51 protein level in BCCIP deficient cells compared to control cells (Figure 9C). This is consistent with a role of BCCIP in HR dependent DSB repair.

### Spontaneous and DNA damage induced structural instability of chromosomes in BCCIP-deficient cells

**Figure 10:**
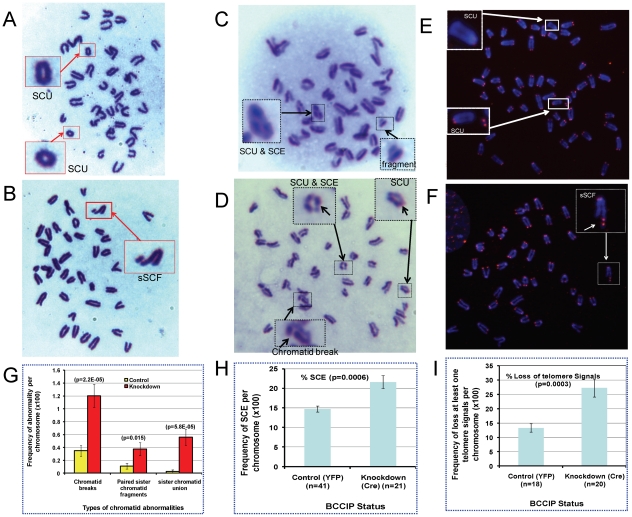
Spontaneous chromosome aberrations in BCCIP-deficient MEFs. The MEF4-LoxPshBCCIP (passage 1) cells with the conditional BCCIP knockdown cassette were infected with control retro-viruses that express YFP, and the Cre-expressing retrovirus to activate the knockdown of BCCIP. Then, the metaphase chromosome spreads (panels A and B), sister chromatid exchange assay (panels C and D), and telomere FISH (panel E and F) were produced (see Materials and Methods) to score chromosome abnormalities. Representative sister chromatid union (SCU), sister chromatid exchanges (SCE), chromatid break with single chromatid fragment (sSCF) Panel B), and chromosome fragments are shown in panels A-F. Panel G showed the frequency of three type of chromatid abnormality in 109 control and 137 BCCIP-knockdown cells. Panel H shows the frequency of sister chromatid exchanges in 41 controls and 21 BCCIP knockdown cells. Panel I illustrates the frequency of loss of telomere signals in 31 controls and 37 BCCIP knockdown cells.

The human BCCIP interacts with BRCA2, and BCCIP deficiency reduces endogenous level of Rad51 (Figure 9). Both BRCA2 and Rad51 are key proteins involved in HR. Under physiological condition, a key function of HR is to resolve stalled replication forks [2], [3], and impaired HR would cause spontaneous structural chromosome alterations. Thus, we investigated whether BCCIP deficiency would cause spontaneous chromosome abnormalities. First, Giemsa-stained chromosome metaphase spreads were prepared from control and BCCIP-deficient MEFs. As represented in Figure 10A–10B, we observed two types of spontaneous chromatid aberrations in BCCIP knockdown MEFs: single chromatid breaks with un-paired chromatid fragments, and SCU (sister chromatid union). We also observed some paired sister chromatid fragments (pSCF) that may be companions with SCU (when the SCU is formed by fusion of telomere-less broken chromatid arms). Figure 10G summarizes the spontaneous frequencies of the types of chromosome abnormalities caused by BCCIP-deficiency. BCCIP-deficiency results in a 3.5-fold increase on single chromatid breaks, and 3.4-fold increase in occurrence of paired sister chromatid fragments. The most dramatic increase is in SCU occurrence. While there was little SCU in the control cells, there was ∼20-fold increase of SCUs in BCCIP knockdown cells. Because the BCCIP knockdown MEF population has more polyploid cells than control MEF, the frequencies of chromosome abnormalities were normalized to the number of chromosomes. The frequency of abnormality, normalized to number of metaphase cells, can be found in Figure S6.

The chromatid break is indicative of failed restart of collapsed replication forks, which generates one-ended DSBs. We further measured whether BCCIP deficiency causes increased sister chromatid exchange (SCE), which is seen in Bloom syndrome and several genetic disorder related to replication stress [31]–[33]. Consistent with the results from Giemsa-stained chromosome metaphase spreads, we observed the induction of SCUs alone with chromatid breaks in BCCIP-deficient cells (Figure 10C and 10D). However, the increase of SCE in BCCIP deficient cells is modest (Figure 10H). This may reflect a potential role of BCCIP in supporting Rad51-dependent strand invasion during the restart of replication forks (see Discussion for details), which is consistent with the observation that BCCIP deficiency causes Rad51 down regulation (Figure 9).

The formation of SCUs is a unique phenotype in BCCIP deficient cells. To our best knowledge, the earliest literature that described this form of chromatid alteration was in 1938 with Drosophila by Kaufmann [34], but has been rarely described since then. We reasoned that SCUs may be produced by two mechanisms: telomere fusion between the sister chromatids; or the re-ligation of the broken sister chromatids. Although the second possibility is suggested by the presence of paired chromatid fragments in the BCCIP deficient cells, telomere FISH was performed to distinguish these possibilities. As can be seen in Figure 10E and 10F, the SCUs were associated with loss of telomere signals and were not caused by telomere fusion. We often observed paired telomere signals from the acentromeric chromatid fragments in the same cells with SCUs. These observations suggest SCU as a consequence of ligation of two broken telomere-less sister chromatids, and unlikely a fusion after telomere erosion. With the same telomere FISH experiments, we observed induction of chromatid breaks with single sister chromatid fragment (sSCF) in BCCIP deficient cells (Figure 10F). We also found an increase in percentage of chromatids that have lost telomere FISH signals (Figure 10I). Altogether, these data (Figure 10) strongly suggest that BCCIP deficiency causes spontaneous chromatid aberrations associated with replication.

**Figure 11:**
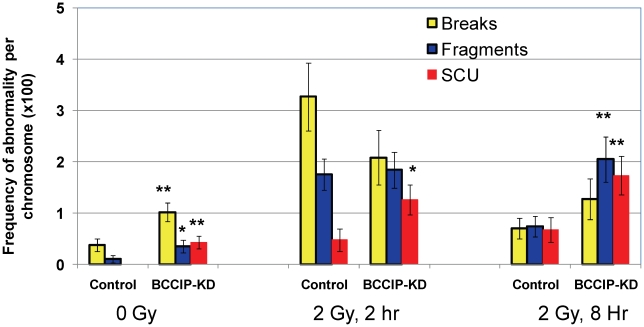
BCCIP deficiency increases radiation induced chromosome aberrations. At 2 and 8 hours after 2 Gy of irradiation, the cells were processed to score major types of chromosome aberrations. Shown are the aberration frequencies. Error bars are standard errors. (*) indicates p<0.05 and (**) indicate p<0.01 when comparing the BCCIP knockdown (BCCIP-KD) and control MEFs at the same dose and time point by student *t*-test.

We further analyzed chromosome abnormalities at 2 and 8 hours after 2 Gy of γ-irradiation. Again, there was a significant increase of spontaneous chromosome abnormalities, including SCU (Figure 11). At 2 hours after the irradiation, SCU frequency is significantly higher in the BCCIP knockdown cells than the control cells, but the other forms of damages are not significantly different between the BCCIP knockdown and control cells. The control cells exhibited significantly less chromosome abnormalities at 8 hours, than at 2 hours after irradiation, indicating repair of DNA damages associated with these forms of abnormalities. However, there remained a significantly higher level of chromosome abnormalities in the BCCIP knockdown cells than the control cells at 8 hours. Noticeably, the SCU level at 8 hours remains as high as at 2 hours, suggesting that the BCCIP deficient cells repaired little damages leading to SCU during the 2–8 hours following irradiation. These data support the notion that BCCIP is not only required to repair different forms of DNA damages but also has a significant role in protecting the cells against SCU.

### p53 deficiency cannot rescue the embryonic lethality in BCCIP-deficient mice

Because BCCIP deficiency spontaneously activates p53 Ser-15 phosphorylation in the MEFs (Figure 6B), and it has been shown that p53 deficiency can partially delay the embryonic lethality conveyed by BRCA1 and BRCA2 deficiency in mice [35], we measured the *in vitro* growth rates of the p53 mutant and wild type BCCIP deficient MEFs. As shown in Figure S7, deletion of p53 can only partially rescue the growth retardation of BCCIP deficient cells. Next we asked whether p53 deficiency can completely rescue the embryonic lethality in BCCIP deficient mice. We used three different strategies to breed the constitutive p53 null mice originally generated by Jacks et al [36]. Table 2 shows the distribution of genotypes among viable newborns after breeding: 1) between (p53+/+;LoxPshBCCIP+/+;EIIaCre−/−) and (p53+/+; LoxPshBCCIP−/−;EIIaCre+/−), 2) between (p53+/−; LoxPshBCCIP +/+;EIIaCre−/−) and (p53+/−; LoxPshBCCIP −/−;EIIaCre+/−), and between (p53+/−; LoxPshBCCIP +/−;EIIaCre−/−) and (p53+/−; LoxPshBCCIP −/−;EIIaCre+/−). As shown in Table 2 (see Table S2 for detailed breeding data), p53 deletion retain approximate 1∶1 ratio between EIIaCre(+/−) and EIIaCre(−/−)mice in LoxPshBCCIP(−/−) background. The EIIaCre(+/−) and EIIaCre(−/−) ratio was significantly less than 1 (16∶86) in p53 wild type mice, and this reduced ratio was not increased in p53 deficient or p53 heterozygous background (0∶24 and 11∶72 respectively). These data suggest that p53 deletion failed to completely rescue the embryonic lethality induced by BCCIP knockdown, suggesting that DNA damage activated p53 signaling cannot fully account for the embryonic death completely.

**Table 2:** Number of viable newborns resulting from crossing LoxPshBCCIP with EIIaCre with or without p53 deletion.

	LoxPshBCCIP(−/−)			
	P53 +/+	p53+/−	p53−/−	Total
EIIaCre −/−	6	31	9	46
EIIaCre +/−	8	32	11	51
Total	14	63	20	97

	LoxPshBCCIP (+/−)			
	P53 +/+	p53+/−	p53−/−	Total
EIIACre −/−	85	72	24	181
EIIACre +/−	16	11	0	27
Total	25	83	24	208

The founder line *LoxPshBCCIP^+/+^-4* was used for this breeding. See text and Table S2 for details.

## Discussion

BCCIP is a BRCA2 interacting protein in human and *Ustilago maydis* [7]–[10], [19]. In this study, we have found that BCCIP deficiency causes chromatid abnormalities especially a dramatic induction of sister chromatid unions (SCUs), and impairs mouse embryo development.

### Defective DNA repair and replication stress in BCCIP-deficient cells

In addition to DSB repair, a major function of the HR machinery is to preserve genomic integrity via resolving replication blockage to reduce replication stress, which is loosely defined as the inefficient progression or stalling of replication forks [3], [37], [38]. During replication, replication forks may be stalled by encountering single-strand breaks or damaged nucleotides that are not by-passed by DNA translesion synthesis. This often produces a one-ended DSB, which can be processed to yield a single stranded 3′-end to initiate a strand invasion and form a single Holliday junction at the stalled replication fork. After branch migration (or replication fork regression) and resolution of the Holliday junction, the stalled replication fork can be re-started. It is believed that many factors of the HR pathway, including BRCA2 and associated proteins, are required in this process.

Replication stress is often manifested by excessive levels of spontaneous single-stranded DNA (ssDNA), or DNA strand breaks. On the cytogenetic level, excessive level of chromatid breaks and SCEs is a signature of replication stress. It has been suggested that endogenous replication stress induced by HR defects may not be detected by the S-phase checkpoint machinery. Thus cells with excessive replication stress can enter mitosis to cause mitotic errors [37]. In a previous report, it was shown that BCCIP deficiency results in accumulation of spontaneous DNA strand breaks and single-stranded DNA in human cells [16]. In this study, we have observed an increase of spontaneous chromatid breaks and SCUs in BCCIP deficient cells (Figure 10), and impaired repair of radiation damages that lead to SCU formation (Figure 11). These results are consistent with a role of BCCIP in suppressing replication stress and repair of DNA damage.

### Sister chromatid exchanges and breaks in BCCIP deficient cells

**Figure 12:**
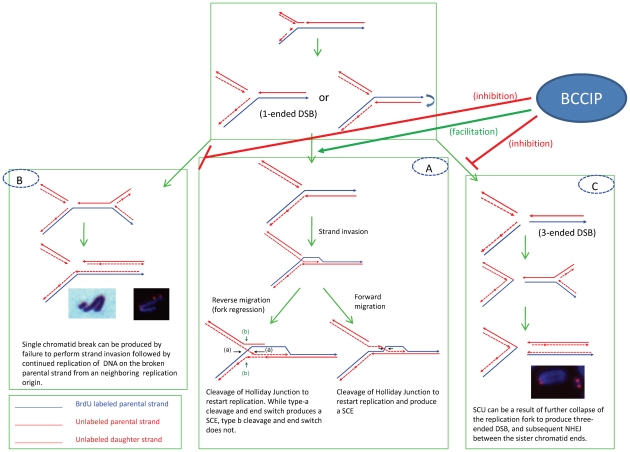
Potential mechanisms to produce SCE (panel A), chromatid breaks (panel B), and SCU (panel C) upon replication fork blockage. BCCIP may function to promote the Rad51-dependent strand invasion (thus SCE) and reduce the probability of further collapse of replication forks to form the 3-ended DNA double strand break ends (panel C) that would lead to the formation of SCU, or permanently form the one-ended DSB structures (panel B) that lead to the formation of single chromatid breaks (see text for more details).

We have observed a significant spontaneous increase in sister chromatid breaks (3.5-fold) yet a modest increase of SCE (∼1.5 fold) in BCCIP deficient cells (Figure 10). These abnormalities have often been used as markers for genomic instability. Although the molecular mechanisms for SCE formation are complex, it is generally believed that the 3′-end of the one-ended DSB of the stalled replication fork initiates the process with strand invasion [31]. Once strand invasion is initiated to form the Holliday junction, branch migration (or fork regression) and resolution of the Holliday junction would produce a SCE (Figure 12A). Therefore, factors that increase the production of one-ended DSBs (e.g. excessive levels of SSBs or inability to carry out translesion synthesis) have the potential to stimulate SCE [31]. Additionally, deficiencies in proteins involved in branch migration of the Holliday junction (e.g. BLM and RecQL5) may favor SCE upon Holliday Junction resolution [31]–[33].

On the other hand, defects in proteins involved in strand invasion may have different consequences on SCE. It has been shown that BRCA2 and RAD51 defects do not significantly increase spontaneous SCE in mammalian cells [31], [39]–[41]. Since strand invasion is a critical step to produce SCE, defective RAD51 and its accessory factors may reduce or only modestly increase SCE due to ineffective strand invasion even in the context of excessive one-ended DSB and replication stress. As a consequence, this would significantly increase chromatid breaks (Figure 12B). In this study, we observed reduced basal level of mouse Rad51 protein and focus formation in the BCCIP deficient cells (Figure 9). This observation is consistent with the increase in chromatid breaks and formation of paired sister chromatids in BCCIP-deficient cells and the reduction of RAD51 focus formation in BCCIP deficient human cells [10], [30]. A possible mechanism for the formation single chromatid breaks is illustrated in Figure 12B.

A question is how BCCIP deficiency may cause reduced Rad51 protein level. It is known that Rad51 preferably expresses in S phase cells. A tempting explanation is that reduction of S-phase cell fraction may reduce the overall Rad51 level in the BCCIP deficient cell population. However, this is unlikely the case because BCCIP deficiency causes replication stress but did not cause overall reduction of S-phase fraction ([11] and data not shown). Although we cannot rule out the possibility that Rad51 protein stability is altered in BCCIP deficient MEFs, we found that the BCCIP deficient MEF cells had reduced Rad51 mRNA level based on RT-PCR analysis (data not shown), suggesting that a down-regulated Rad51 transcription may contribute to the Rad51 protein level.

### SCU is a unique phenotype in BCCIP-deficient cells

A characteristic structural chromosome alteration in the BCCIP deficient MEFs is SCU, which is not only induced spontaneously but also remains high 2–8 hours after irradiation (Figure 10 and Figure 11). The telomere FISH experiments have suggested that SCUs are likely the consequence of ligation between two broken sister chromatids. We envision that SCU may be caused by the following scenario (Figure 12C). When one-ended DSB resection and subsequent strand invasion fails, an excessive level of single sister chromatid breaks and further collapse of the replication fork result in three one-ended DSBs (as shown in Figure 12C). Then, SCU may occur upon re-ligation of the sister chromatid DSB ends. The proximate DNA fragment may resume replication due to the presence of multiple replication origin sites. This produces paired chromatid fragments. However, we would like to emphasize that alternative mechanisms to produce SCU are possible. For example, late S-phase cells with failed resolution of HR intermediates and/or replication termination structures may form DSB on sister chromatids, thus SCU. Although erosion of telomeres in telomerase deficient cells may expose the chromtid ends to form SCU, this scenario is unlikely to be the cause of SCU in BCCIP deficient MEF cells, because the BCCIP deficient cells were cultured in vitro for only a few passages. It would be interesting to investigate whether eroded telomere ends can form SCU in *Tert* deficient cells after long-term culture.

Nevertheless, the SCUs will likely form chromatin bridges between daughter nuclei at anaphase. It is expected that this form of structural abnormality will result in chromosome segregation errors and numerical chromosome instability in daughter cells.

### Embryonic lethality of BCCIP-deficient mice

The phenotypes of BCCIP deficient embryos are consistent with BCCIP's orthologs in lower eukaryotes, and its interaction partner BRCA2 [35]. Several BRCA2 knockout mouse models have been developed. Depending on the specific regions deleted in the knockout model, the embryonic phenotype of BRCA2 mutant mice varies [35]. However, most mouse models with large deletions on BRCA2 produce embryonic lethality [35]. In this study, we have established a LoxP-Cre based conditional BCCIP knockdown mouse model. Using this model, we have shown that the mouse *BCCIP* gene is essential for embryonic development. Although many mechanisms may contribute to embryonic abnormality of *BCCIP*-deficient mice, the accumulation of spontaneous DNA damage, excessive replication stress, and formation of lethal chromatid aberrations in *BCCIP* deficient cells are considered the major initiating factors. Down regulation of *BCCIP* has been shown to cause spontaneous DNA damage in human cells [16]. In this study, we observed spontaneous activation of p53 together with up-regulation of p21 in BCCIP deficient MEFs (Figure 6B), and increased cell death through apoptosis at day E7.5 follows reduction in cell proliferation (Figure 5). These observations are consistent with the scenario that BCCIP deficiency leads to accumulation of spontaneous DNA damage, thus growth inhibition and cell death, which lead to embryonic lethality. However, the accumulation of spontaneous DNA damage along with activation of p53 may not fully account for embryo lethality, as the p53 deletion did not completely rescue the embryonic lethality of BCCIP deficiency despite that it can partially rescue the growth retardation of BCCIP deficient MEFs *in vitro* (Figure S7). Second, BCCIP deficiency may inhibit proliferation by disrupting cell division in mitosis. We found that BCCIP-deficient cells had significantly increased levels of spontaneous chromatid breaks and SCUs at metaphase (Figure 10). It is anticipated that the chromatid breaks will cause a net loss of chromosomal materials after mitosis, and the SCUs will evolve into chromatid bridges at anaphase and telophase to disrupt chromosome segregation, both scenarios are potentially lethal to the cells and can contribute to the embryo development defects.

### Conditional knockdown as an effective and alternative approach to conventional knockout

The conditional knockdown approach offers advantages over conventional knockout approach. It may avoid interference with the overlapping genes. Second, while the conventional knockout approach would only offer either homozygous or heterozygous gene ablation, the knockdown approach may grant us the ability of mimicking abnormal protein expression that might occur in human diseases. Down regulation of BCCIP has been shown in cancers [7], [9], [13], [14]. Considering the strong genomic instability phenotype in BCCIP deficient cells and the multiple functions of BCCIP [10], [11], [15]–[18]. It is likely that BCCIP deficiency may contribute to tumorigenesis in mice. Because the EIIa-Cre mediated BCCIP knockdown causes embryonic lethality, tissue specific conditional knockdown is in process to address whether BCCIP down-regulation contribute to tumorigenesis.

In summary, our study suggests a critical role of mouse BCCIP gene in maintaining genomic stability and embryonic development. The formation of characteristic sister chromatid union in BCCIP deficient cells may reflect a unique molecular function of BCCIP in resolving stalled replication forks, and may contribute significantly to embryonic development defects.

## Materials and Methods

### Ethics statement

The animal works presented in this study were approved by Institutional Animal Use and Care Committee of Robert Wood Johnson Medical School-UMDNJ. We follow our institutional guideline regarding to animal welfare issues.

### Generation of conditional transgenic mice expression shRNA against the mouse BCCIP

The pBS/U6-pLoxPneo vector [23] was kindly provided by Dr. Chuxia Deng (National Institute of Diabetes and Digestive and Kidney Disease, NIH). A pair of mouse BCCIP specific oligonucleotide (5′-GGATGAAGATGAGATCTTTGGTTCAAGAGACCAAAGATCTCATC TTCATCCTTTTTT-3′ and 5′-AATTAAAAAAGGATGAAGATGAGATCTTTGGTCTCTTGAACCAAAGATCTCATCTTCATCCGGCC-3′) were annealed, and then ligated into the pBS/U6-pLoxPneo vector digested with ApaI and EcoRI. This results in the conditional mouse BCCIP knockdown vector designated *pBS/U6-pLoxPneo-shBCCIP*. The effectiveness of this vector to knockdown mouse BCCIP was confirmed by stably transfecting vectors into mouse NIH3T3 cells, and then transiently expressing the Cre recombinase in the cells.

The conditional BCCIP knockdown vector (pBS/U6-pLoxPneo-shBCCIP) was digested by KpnI and NotI. The linearized 2.3 kb DNA fragment containing the conditional LoxPshRNA expression cassette was injected into pronuclei of fertilized oocytes isolated from superovulated FVB/N mice. Then the injected oocytes were implanted into pseudopregnant recipient females. Genomic DNA was extracted from tail biopsies of the resulting litters and analyzed by PCR and Southern blot. Among the 27 mice obtained from the injections, 7 were found to be positive for the LoxPshBCCIP transgene cassette. The U6-LoxP-shBCCIP positive mice were crossbred with FVB/N wild type mice to identify the mouse lines capable of germline transmission. Through this procedure, two founder lines with high germline transmission were identified. They were designated as *LoxPshBCCIP-4* and *LoxPshBCCIP-13*, and both were successfully bred into homozygsity (*LoxPshBCCIP^+/+^*). By breeding with wild type mice, the homozygous transgenic mice (*LoxPshBCCIP^+/+^*) is distinguished from heterozygous mice (*LoxPshBCCIP^+/−^*) because the homozygous transgenic mice are able to produce 100% of LoxPshBCCIP positive newborns while the heterozygous mice (*LoxPshBCCIP^+/−^)* can produce only 50% of *LoxPshBCCIP* positive mice. The PCR primer pairs used for genotyping were: 5′-TCTAGAACTAGTGGATCCGAC -3′, and 5′-TCGTATAGCATACATTATACG-3′. The probe used for Southern blot was generated by a PCR amplification of the conditional knockdown vector using the following primers: 5′-ATTGAACAAGATGGATTGCACGCA, and 5′-TCAGAAGAACTCGTCAAG AAGG-3′.

### Cross-breeding of conditional BCCIP knockdown mice with EIIa-Cre transgenic mice

The homozygous FVB/N-Tg (EIIaCre^+/+^) C5379Lmgd/J mice (Lakso, 1996), were purchased from Jackson Laboratory (stock number: 003724), and crossed with wild type FVB/N mice to obtain EIIaCre^+/−^ heterozygous mice. Then the LoxPshBCCIP^+/+^ homozygous mice were bred with the EIIa-Cre^+/−^ heterozygous mice. Theoretically, this will generate offspring with two genotypes: [LoxPshBCCIP^+/−^;EIIaCre^+/−^] with BCCIP knockdown, and [LoxPshBCCIP^+/−^; EIIaCre^−/−^] as a control at a 1∶1 ratio. If the knockdown of BCCIP is lethal during embryogenesis, reduced newborn ratio of [LoxPshBCCIP^+/−^; EIIaCre^+/−^] to [LoxPshBCCIP^+/−^; EIIaCre^−/−^] is anticipated. The PCR primers to genotype EIIaCre were 5′CCTGTTTTGCACGTTCACCG3′ and 5′ATGCTTCTGTCCGTTTGCCG3′, which results in a PCR product of ∼270 bp. The animal works were approved by Institutional Animal Use and Care Committee of Robert Wood Johnson Medical School.

### Antibodies, Western blot, and immuno-fluorescent staining of cultured cells

To generate rabbit anti-mouse BCCIP antibodies, mouse cDNA coding for C-terminal 292aa was cloned into pET28 vector (Novagen, Madison, WI). Recombinant (6×His)-tagged mouse BCCIP protein was expressed and purified with BL21 (DE3) cells, and the GST-mouse BCCIP protein was expressed and purified in BL21 cells using pGEX vector as previously described [42], [43]. The HIS-tagged BCCIP was injected into rabbits to produce polyclonal antibodies, and GST-mouse BCCIP was used for affinity purification of polyclonal anti-BCCIP antibodies. Anti- PCNA (PC-10), p21 (F-5), p53 (FL-393), and c-myc monoclonal antibody were purchased from Santa Cruz Biotechnology (Santa Cruz, CA). Anti- γH2AX, phospho-p53 (Ser15) and anti-cleaved caspase-3 antibodies from Cell Signaling (Danvers, MA); anti-pericentrin antibody from Covance Research Products Inc (Berkeley, CA), anti-γ tubulin antibody from Sigma (St, Louis, MO), and anti-Brachyury and anti-Ki67 from Abcam (Cambridge, MA). Western blots were performed with procedures as described previously [7], [11], [15], [16], [18], [44].

### Histological analysis and BCCIP immunohistochemistry

Uteri from female mice were isolated at days E6.5–8.5, the individual decidual swellings were isolated transversely according to the methods of Smith (Smith, 1985), rinsed with cold PBS, fixed overnight in 4% paraformaldehyde at 4°C, then embedded in paraffin. Serials of 5 µm sections were cut and stained with hemotoxylin and eosin. Anti-BCCIP polyclonal antibody (1∶100), anti-Ki67 polyclonal antibody (1∶300), anti-cleaved caspase3 polyclonal antibody (1∶100), and anti-Brachyury (1∶100) antibodies were used for immuno-histochemical staining of the corresponding proteins using previously developed protocols [20].

### *In situ* detection of incorporated BrdU by immunohistochemistry

To measure DNA synthesis in embryo mouse tissues, BrdU (100 µg/g of body weight) was intraperitoneally injected into pregnant female mice. One hour later, the entire uteri were removed, and the individual decidual swellings were isolated, fixed in 4% paraformaldehyde at 4°C overnight, embedded in paraffin, and sectioned (5 µm). To stain incorporated BrdU, the sections were de-paraffinized, treated with 2 N HCl for 30 min at 37°C, incubated with anti-BrdU monoclonal antibody (Becton Dickinson, Franklin Lakes, NJ) at a 1∶500 dilution for 2 hr at 37°C, and then incubated with anti-mouse-HRP secondary antibody for 1 hr. 3,3′-Diaminobenzine tetrahydrochloride hydrate (DAB) color developed. BrdU positive cells are visualized by their brown color with DAB, and BrdU negative cells display blue color by hematoxylin.

### TUNEL assays with tissue sections

Paraffin embedded tissue sections (5 µm) were used to detect apoptotic cells using DeadEnd Fluorometric TUNEL System (Promega, Madison, WI). Briefly, sections were rinsed 3 times with distilled H_2_O, once in PBS, and permeabilized with 200 µg/ml of Proteinase K in PBS for 15 min. The permeabilized sections were incubated with equilibration buffer for 10 min at room temperature. DNA strand-break labeling and colorization were performed according to the manufacturer recommended procedures, mounted with VECTASHIELD fluorescent mounting media with DAPI, and the results were recorded with fluorescent microscope.

### *In vitro* culture of pre-implantation blastocysts

Homozygous BCCIP female FVB/NJ mice (3.5–4 week old) were given 5 IU of pregnant mare's serum gonadotropin by intraperitoneal injection between 3–4 pm. At 46–48 h post-PMSG injection, they were treated with 5.0 IU of human chorionic gonadotrophin (hCG) by intraperitoneal injection, and then mated with wild type and EIIaCre^+/+^ male mice individually. Next morning, the female mice with positive mating-plugs were separated from male mice. At embryo day 3.5 (E3.5), blastocysts were collected by flushing the uteri of female mice, and individually cultured for 5 days in 24-well plates in ES cell culture media without leukemia inhibitory factor (Liu, 1996, Suzuki, 1997) with 5% CO_2_ at 37°C. The growth of the cultured blastocysts was monitored daily and photographed.

### Establishment of primary mouse embryo fibroblast (MEF) culture

Primary mouse embryo fibroblast (MEF) cells were generated from day 13.5–14.5 embryos of LoxPshBCCIP^+/+^ female mice (founder line 4) mated with LoxPshBCCIP^+/+^ homozygous male mice according to the protocols by Hertzog [45]. The MEF cells were counted and plated into 10 cm dishes at a density of 0.5–1×10^5^ per cm^2^ in DMEM medium containing 10% FBS, and incubated at 37°C with 5% CO_2_. After 24 hr, the medium, cellular debris, and any unattached cells were removed. The attached MEF cells were designated as passage 0. After 2–3 days of culture, each 10 cm plate of cells was split into 3–5 of 10 cm plates, and the split cultures were then designated as MEF cell passage 1. All *in vitro* experiments, except specifically noted, were carried out with the first passage MEF cells.

### Cell culture and retrovirus infection

A retroviral packaging cell line specific for mouse cell lines (ϕEco), and mouse embryo fibroblast cells (MEF), were cultured in DMEM medium supplement with 10% fetal bovine serum, 100 U/ml of penicillin, 100 µg/ml of streptomycin, and 1% of glutamine. The ϕEco cells were transfected with pLXSP-YFP and pLXSP-myc-Cre retrovirus vector separately. Forty eight hours after transfection, transfected cells were selected by puromycin (1 µg/ml) for 2 days. Then the cells were grown to 80–90% confluence in regular culture medium. Virus suspensions were collected, filtered with 0.45 sterile syringe filters, and mixed with 8 µg/ml of polybrene (Sigma, St, Louis, MO). The MEF cells were infected 3-times with the virus during a 2 day period, and then selected with 2.5 µg/ml puromycin for 2 days prior to phenotype analyses.

For cell growth analysis, cells were counted using a Coulter counter (Beckman Coulter, Fullerton, CA). Cells were initially seeded onto 6 cm dish at a density of 0.1×10^6^ per dish, then cell number was determined daily for the next 5 days after the initial plating. Triplicates for each group at each time point were used in the measurements.

### Replication recovery after Aphidicolin (APH) treatment

To assess the ability of MEFs to recover from replication blockage, 0.1×10^6^ MEF cells were grown on 18 mm cover slides in 6-well plate. Cells were treated with 0.4 µM APH in DMEM media for 37°C for 30 hours. After removing APH containing media by rinsing with sterile PBS, the recovery of replication of was measured by measuring Bromodeoxyuridine (BrdU) incorporation. Briefly, BrdU was added to each well to a concentration of 10 µM and slides were fixed at 0, 2, 3, 4, and 5 hours after adding BrdU using 4% paraformaldehyde for 10 minutes at room temperature. The fixative was removed by washing the cover slides three times with 1× PBS. The slides in the wells were treated with 1 M HCl for 10 min in ice, 10 min at room temperature, and 40 min at 37°C to denature DNA. Acid was removed and neutralized by washing the cover slides three times with borate buffer (pH 8.5). Cover slides were then washed three times in PBS+ 0.05% Tween 20 [PBS/T20], blocked with 1 ml of PBS/T20/2% normal goat serum at 37°C for 30 minutes. Cells were immuno- stained with mouse anti-BrdU antibody (1∶200 in 0.1 ml of PBS/T20/2% normal goat serum) and incubating at room temperature for 1 hour or at 4°C overnight. The cells were washed three times with PBS+ 0.05% Tween-20 and stained with donkey anti-mouse Rhodamine conjugate diluted to 1∶500 in 0.1 ml PBS+ 0.05% Tween-20 with 3% BSA and incubated at room temperature for 1 hour. Cover-slides were washed three times with PBS/T20, and mounted on glass slides using Vecta shield+DAPI mounting media. Slides were evaluated using immunofluorescent microscopy and the percentage of BrdU positive cells were counted by counting BrdU positive and DAPI stained cells on each slide.

### Chromosome analysis

To prepare metaphase chromosome spreads (Brown, 2000; Ko, 2008), cells at 80–90% confluence were subcultured into fresh medium, and incubated at 37°C for 24 hours. Colcemid (Sigma, St. Louis, MO) was added at final concentration of 0.2 µg/ml and incubated at 37°C for 4 hours. Cells were trypsinized, and suspended in 75 mM KCl hypotonic solution at 37°C for 15 minutes, and then fixed in fresh 3∶1 methanol/acetic acid. After 3–4 times of additional fixation, suspended cells were dropped onto cold wet slides, allowed to dry at room temperature, and stained with 1% Giemsa. At least 50 metaphase cells were analyzed under 1000× magnification with microscope for each group. Gross chromosome aberrations were scored. Statistical analyses for frequency of aberrations were performed using *t-test*, and a P value of <0.05 was considered significant.

### Telomere FISH analysis

The methods developed by Williams et al were used [46], [47]. In brief, after the MEF cells were subcultured into fresh medium and cultured for 24 hours, 0.2 µg/ml Colcemid was added for 6 hours to accumulate mitotic cells. Cells were trypsinized with Trypsin-EDTA (Gibco, Carlsbad, CA) and suspended in 75 mM KCl hypotonic solution at 37°C for 15 minutes before fixation. After four times of repeated fixation in fresh 3∶1 methanol/acetic acid, cells were dropped onto cold slides and allowed to dry slowly in a humid slide box. A probe to telomeric DNA was prepared by synthesizing an oligomer having the sequence (CCCTAA)_7_ and was labeled by terminal deoxynucleotidal transferase tailing (Roche, Florence, SC) with SpectrumRed-dUTP (Vysis, Des Plaines, IL) according to the manufacturer's instructions. A hybridization mixture containing 0.4 µg/ml probe DNA in 30% formamide and 2×SSC (1×SSC is 0.15 M NaCl, 0.015 M sodium citrate) was applied to slides that had been denatured in 70% formamide, and 2× SSC at 70°C for five minutes. Following an overnight hybridization at 37°C in a moist chamber, the slides were washed in 2× SSC at 42°C (5 times, 15 min each) twice, and then placed in PN Buffer (100 mM Na2HPO4, 50 mM NaH_2_PO_4_, 0.1%Triton X-100) at room temperature for 5 minutes and mounted in fluorescence mounting medium with DAPI. Metaphase cells examined with a Zeiss fluorescence microscope and images were captured with HAL100 camera. At least 20 metaphase cells were analyzed for each group. Chromosome aberrations (breaks, fragments, and sister chromatid union) were scored. Statistical analyses for frequency of aberrations were performed using *t-test*.

### SCE (sister chromatid exchange) analysis

The method developed by Wang et al. was modified [48]. The MEF cells were cultured with medium containing 10 µM bromodeoxyuridine (BrdU) for 24 hour and then cultured in growth medium for another 24 hour, and treated with 0.2 µg/ml of colcemid 6 h before collection. The harvested cells were treated with 75 mM KCl hypotonic solution at 37°C for 15 minutes, and fixed with fresh 3∶1 methanol/acetic acid. The cell suspension were dropped onto slides and air-dried. The slides were incubated with 10 µg/ml Hoechst 33258 in ddH_2_O for 20 min, and rinsed with MacIlvaine solution (164 mM Na_2_HPO4, 16 mM citric acid pH 7.0) for three times. The slides were mounted in MacIlvaine solution, and exposed to UV light for 45 min. After washing with PBS for 3 times, the slides were incubated in 2× SSC (0.3 M NaCl, 0.03 M sodium citrate) solution at 62°C for 1 hour, and then stained with 1% Giemsa solution at pH 6.8 for 20 min. Metaphase cells were examined with Olympus microscope and images were captured with PictureFrame. At least 20 metaphase cells were analyzed for each group. Statistic analyses for frequency of aberrations were performed using *t-test*.

### Breeding of p53 mice with BCCIP conditional knockdown mice

The heterozygous p53 knockout mice [36] were crossed with *LoxPshBCCIP^+/+^-4* mouse and EIIaCre^+/+^ mouse respectively to generate *p53^+/−^;LoxPshBCCIP* ^+/−^, and *p53^+/−^;EIIaCre* ^+/−^ mice. The PCR primers used to genotype p53 are: p53ex6F: 5′-GTATCCCGAGTATCTGGAAGACAG-3′, p53neoF: 5′-GCCTTCTATCGCCTTCTTGACG-3′, p53ex7RN: 5′-AAGGATAGGTCGGCGGTTCATGC-3′. The same PCR primer pairs as described earlier in this report were used for BCCIPshRNA and EIIaCre genotyping. The *p53^+/−^;LoxPshBCCIP* ^+/+^ mice were obtained by crossing *p53^+/−^;LoxPshBCCIP* ^+/−^ females with *p53^+/−^;LoxPshBCCIP* ^+/−^ males. The *p53^+/−^;LoxPshBCCIP* ^+/+^ or *p53^+/−^;LoxPshBCCIP* ^+/−^ mice were crossed with *p53^+/−^;EIIaCre* ^+/−^ mice respectively.

## Supporting Information

Figure S1 Western blot confirming the effectiveness of Cre-mediated BCCIP knockdown in the mouse embryo fibroblast (MEF) from two conditional founder mouse lines. MEF cells were established from the conditional knockdown mouse founder lines 4 and 13. Then the MEF were infected with adenovirus that express Cre recombinase, and Western blot performed 3 days after the infection. As can be seen here, the mouse BCCIP protein can be efficiently knocked down by expression of Cre, with line 4 (Panel A) exhibiting a slightly better knockdown efficiency than line 13 (panel B). Lane 1: no infection; Lane 2: control virus- no Cre expression(1∶500); and Lane 3: Cre-expressing virus (1∶500).
(TIF)

Figure S2 Genotyping and BCCIP expression in embryos resulted from breeding between LoxPshBCCIP^+/+^ (founder line-13) and EIIaCre^+/−^. At day E11.5, the mouse embryos were dissected, individual embryos are photographed, and shown in Panel A (number 1–4 are abnormal embryos, and number 5–9 are normal). Then, half of each embryo was used to extract DNA for genotyping the conditional shRNA expression cassette that is present in all embryo (panel B1), and the Cre-expressing cassette (panel B2) that is only present in the abnormal embryos. The other half was used to extract the total proteins, which were used to detect mouse BCCIP expression (panel C1). Anti-actin blot (panel C2) was used as a loading control.
(TIF)

Figure S3 Immunohistochemical analysis of Brachyury expression (E6.5–7.0) in wild-type and BCCIP mutant embryos. A: wild-type embryos; B: BCCIP mutant embryos. Arrow point out dark brown brachyury positive cells in primitive streak and mesoderm in wild-type embryos, no expression is detected in mutant embryos.
(TIF)

Figure S4 Growth inhibition of control and BCCIP deficient MEF cells by different doses of γ-irradiation. Control and BCCIP knockdown MEF cells were irradiated with 1–4 Gy of γ-rays. Three days after the irradiation, the number of viable cells were counted, and normalized to the group without irradiation. Shown are the relative numbers of viable cells at the time of analysis.
(TIF)

Figure S5 Representative images of immuno-fluorescent staining of γH2AX at indicated time points. See Figure 8A and 8B for quantification.
(TIF)

Figure S6 Spontaneous chromosome aberrations in BCCIP deficient MEFs. Shown are the frequencies of three types of chromatid abnormalities in 109 controls and 137 BCCIP-knockdown cells.
(TIF)

Figure S7 Partial rescue of growth retardation by p53 deletion in BCCIP deficient MEFs. Panel A shows the growth curves of BCCIP wild type (YFP) and knockdown (Cre) MEFs with wild type p53 (red lines) or null p53 (black lines). Data are average of 3 independent measurements, and each measurement had three replicas. As shown here, the p53 deletion resulted in a better growth in both control (YFP) and BCCIP knockdown (Cre) MEFs. However, with the p53 null background, the Cre-expressing (BCCIP knockdown) cells still grew slower compared to the controls. Panel B shows the ratios of cell numbers between p53 null and p53 wild type control (black line) and BCCIP deficient (red line) cells. As can be seen here, this ratio is higher for BCCIP deficient MEFs than that of the YFP control MEFs, suggesting a preferred stimulation of cell growth by null p53 in the BCCIP deficient cells than the control.
(TIF)

Table S1 Number of blastocysts analyzed.
(DOC)

Table S2 Number of viable newborns obtained from three different breeding strategies.
(DOC)
