# Supplementary figures and images for: Essential Roles of BCCIP in Mouse Embryonic Development and Structural Stability of Chromosomes
Source: PLoS Genet. 2011 Sep 22;7(9):e1002291. doi: 10.1371/journal.pgen.1002291 (PMC3178617; doi:10.1371/journal.pgen.1002291)

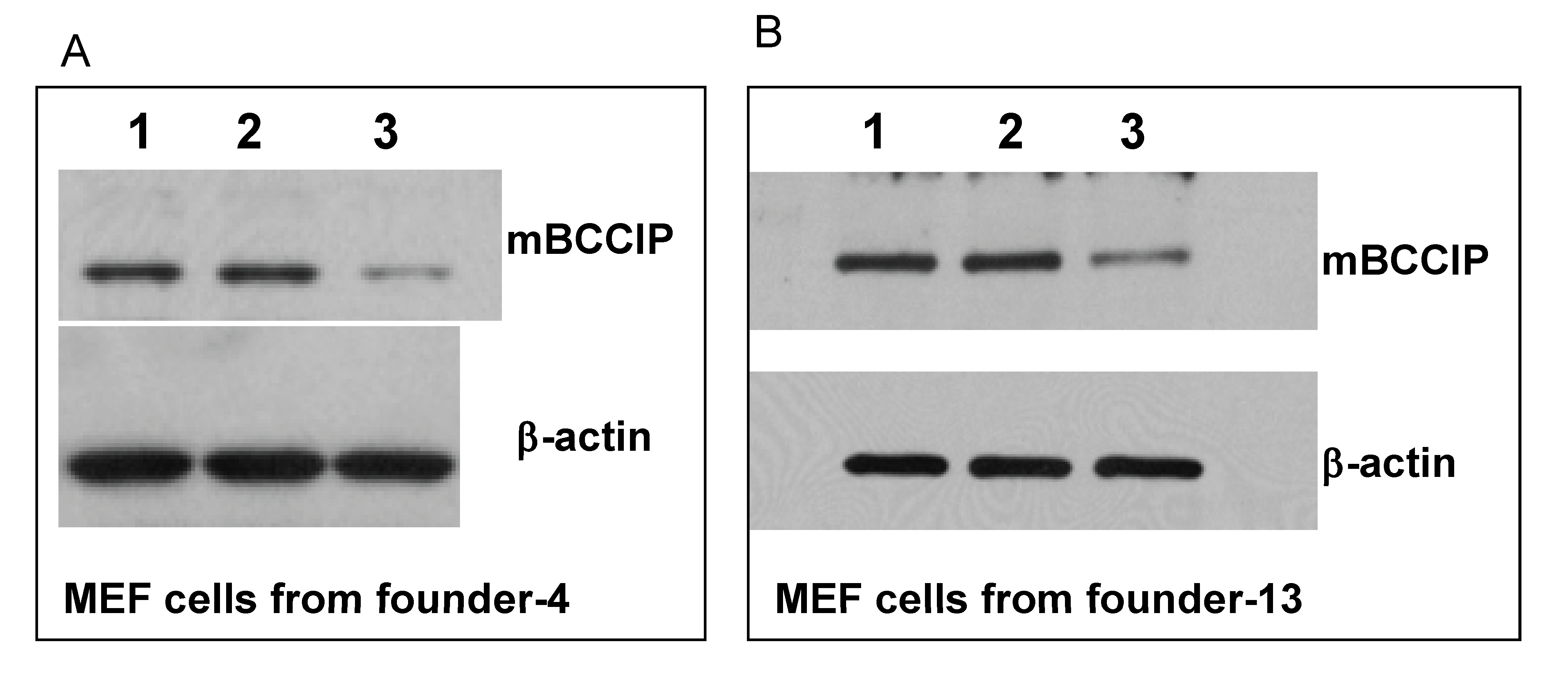

Supplement: Figure S1 — Western blot confirming the effectiveness of Cre-mediated BCCIP knockdown in the mouse embryo fibroblast (MEF) from two conditional founder mouse lines. MEF cells were established from the conditional knockdown mouse founder lines 4 and 13. Then the MEF were infected with adenovirus that express Cre recombinase, and Western blot performed 3 days after the infection. As can be seen here, the mouse BCCIP protein can be efficiently knocked down by expression of Cre, with line 4 (Panel A) exhibiting a slightly better knockdown efficiency than line 13 (panel B). Lane 1: no infection; Lane 2: control virus- no Cre expression(1∶500); and Lane 3: Cre-expressing virus (1∶500). (TIF) [file pgen.1002291.s001.tif]

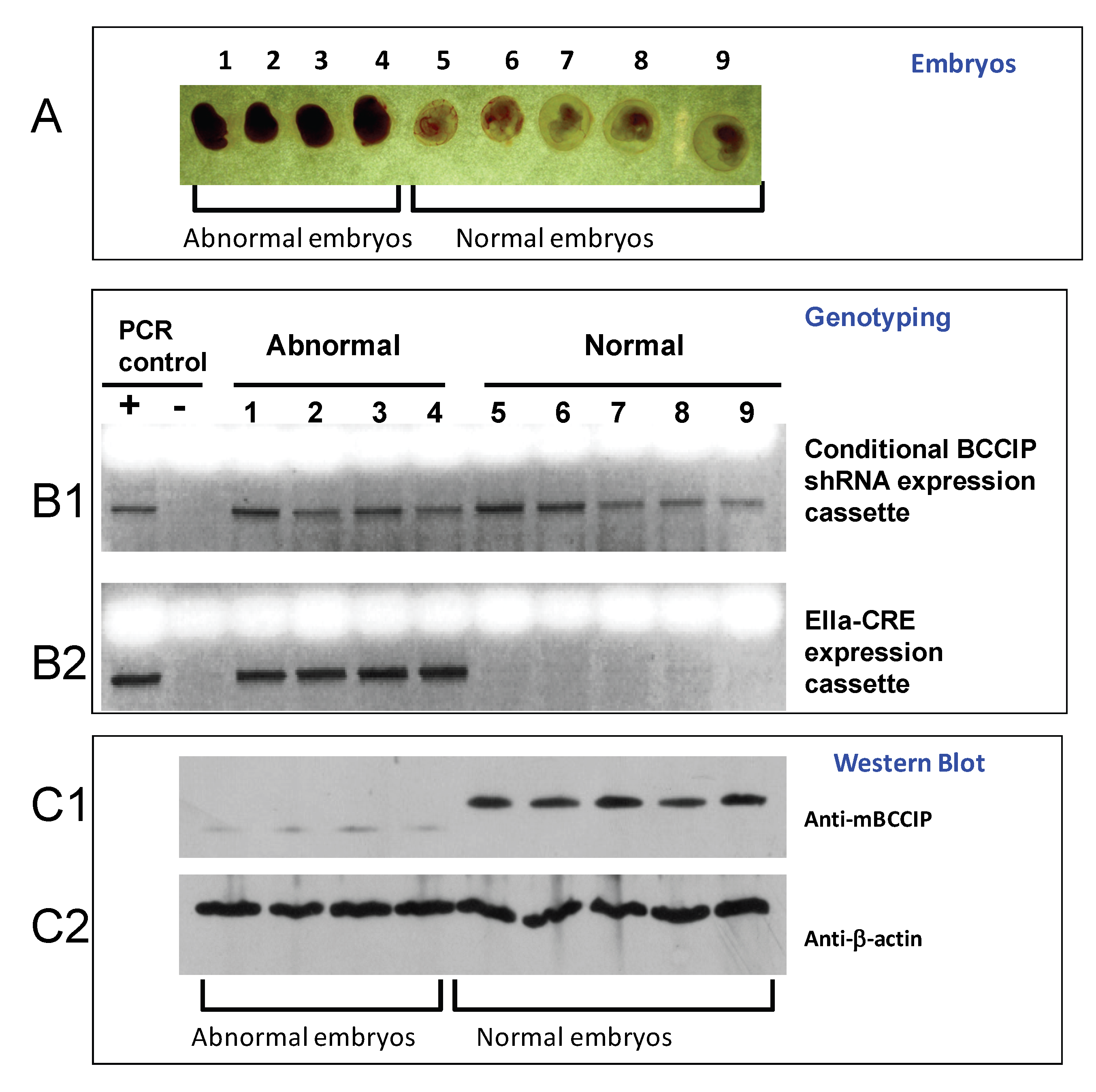

Supplement: Figure S2 — Genotyping and BCCIP expression in embryos resulted from breeding between LoxPshBCCIP+/+ (founder line-13) and EIIaCre+/−. At day E11.5, the mouse embryos were dissected, individual embryos are photographed, and shown in Panel A (number 1–4 are abnormal embryos, and number 5–9 are normal). Then, half of each embryo was used to extract DNA for genotyping the conditional shRNA expression cassette that is present in all embryo (panel B1), and the Cre-expressing cassette (panel B2) that is only present in the abnormal embryos. The other half was used to extract the total proteins, which were used to detect mouse BCCIP expression (panel C1). Anti-actin blot (panel C2) was used as a loading control. (TIF) [file pgen.1002291.s002.tif]

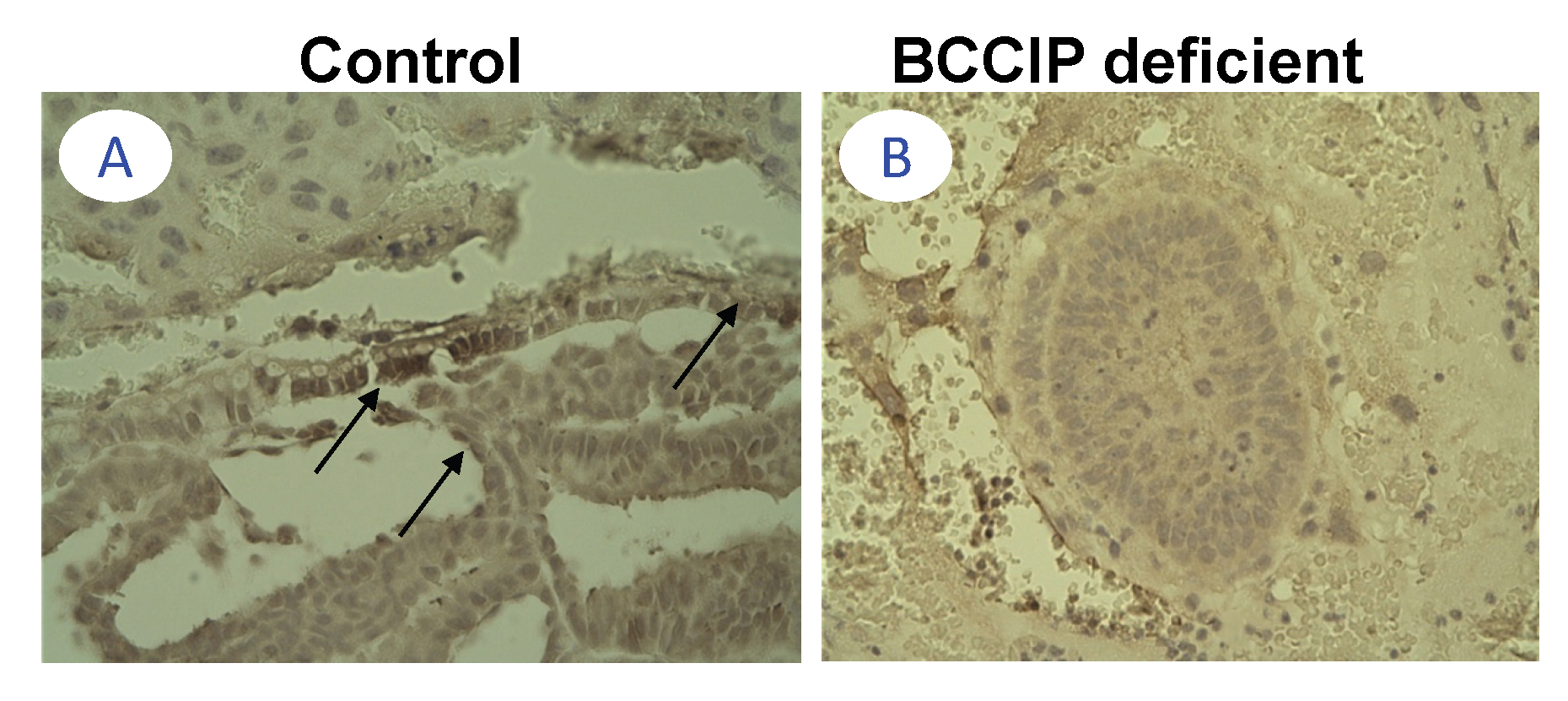

Supplement: Figure S3 — Immunohistochemical analysis of Brachyury expression (E6.5–7.0) in wild-type and BCCIP mutant embryos. A: wild-type embryos; B: BCCIP mutant embryos. Arrow point out dark brown brachyury positive cells in primitive streak and mesoderm in wild-type embryos, no expression is detected in mutant embryos. (TIF) [file pgen.1002291.s003.tif]

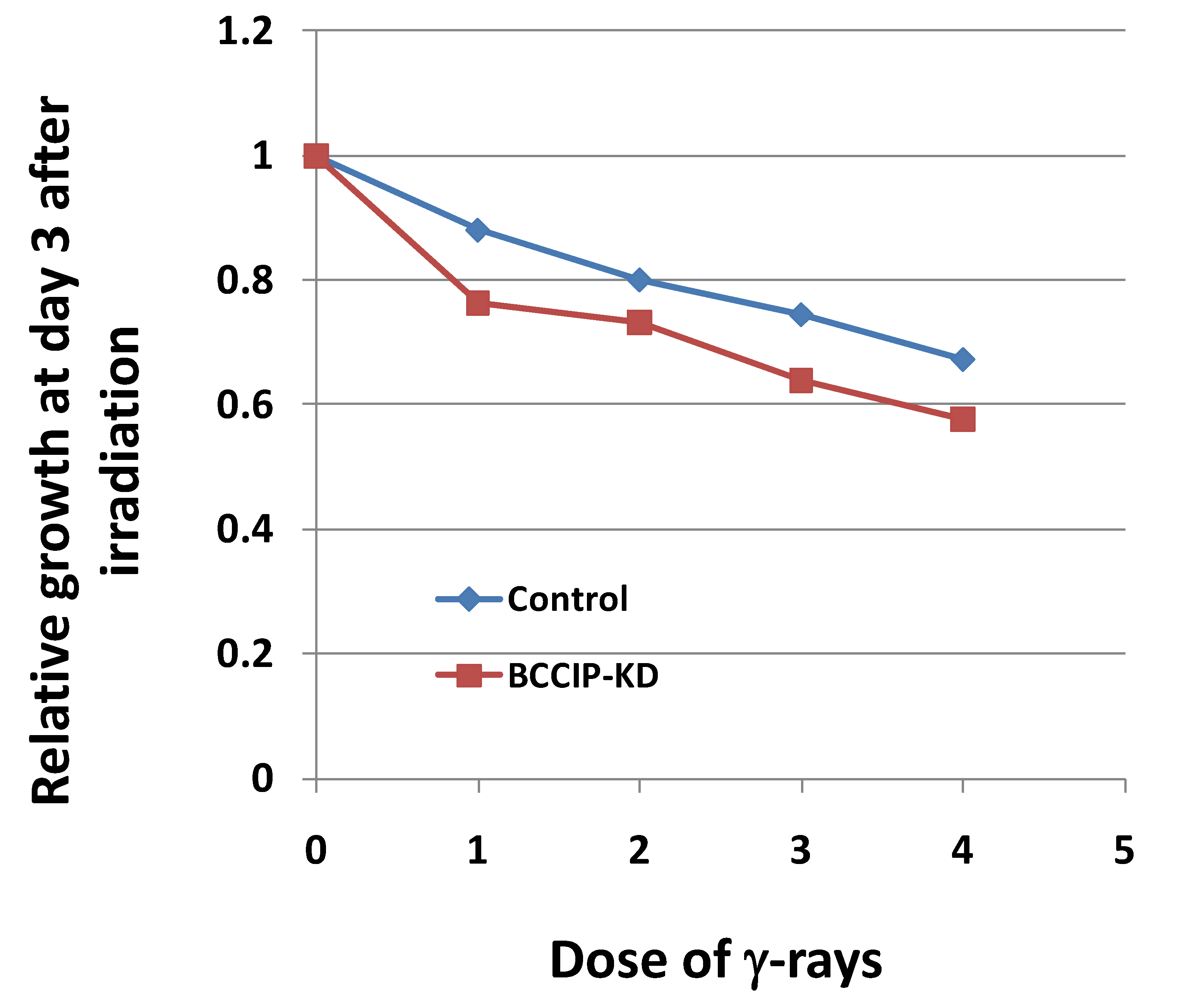

Supplement: Figure S4 — Growth inhibition of control and BCCIP deficient MEF cells by different doses of γ-irradiation. Control and BCCIP knockdown MEF cells were irradiated with 1–4 Gy of γ-rays. Three days after the irradiation, the number of viable cells were counted, and normalized to the group without irradiation. Shown are the relative numbers of viable cells at the time of analysis. (TIF) [file pgen.1002291.s004.tif]

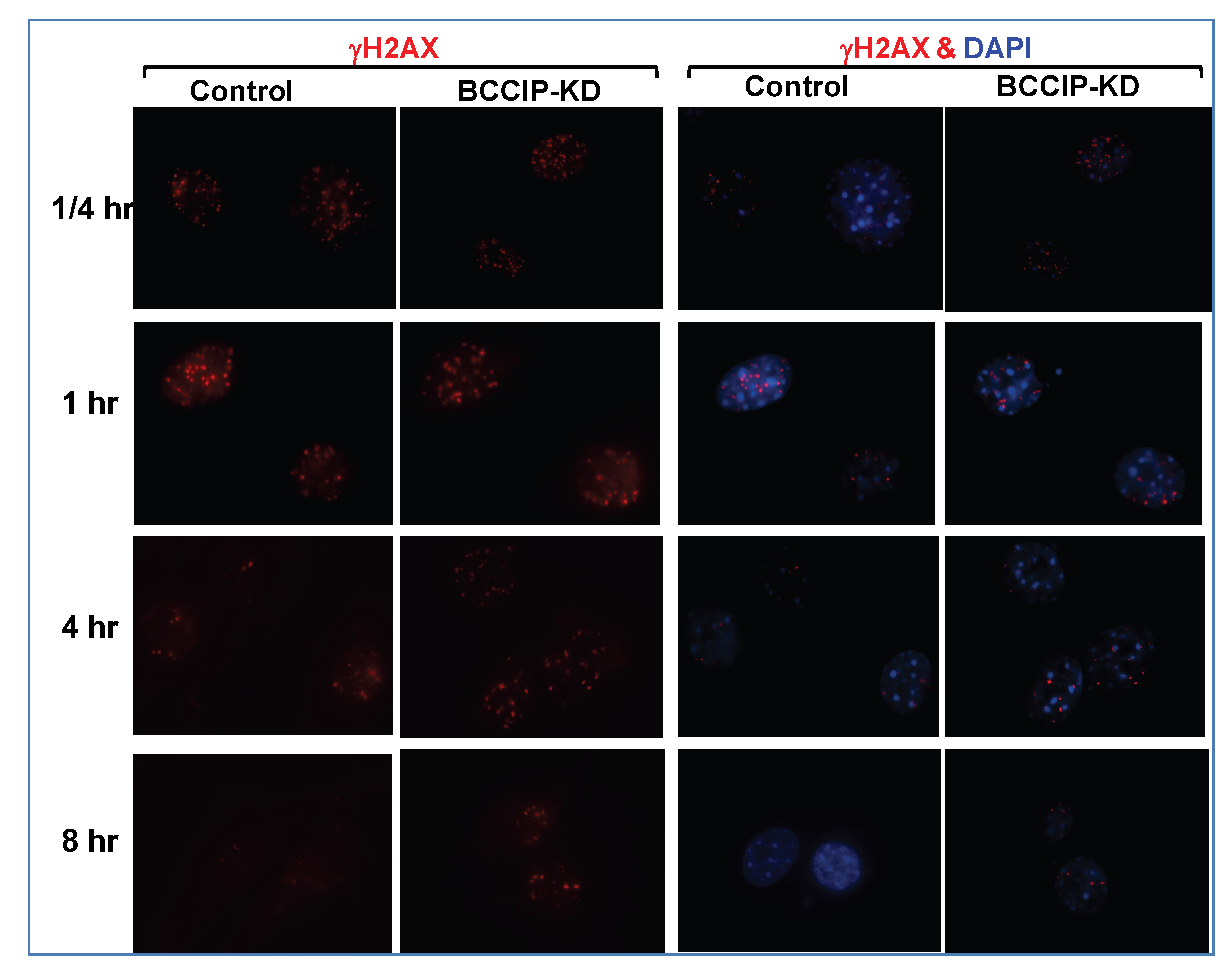

Supplement: Figure S5 — Representative images of immuno-fluorescent staining of γH2AX at indicated time points. See Figure 8A and 8B for quantification. (TIF) [file pgen.1002291.s005.tif]

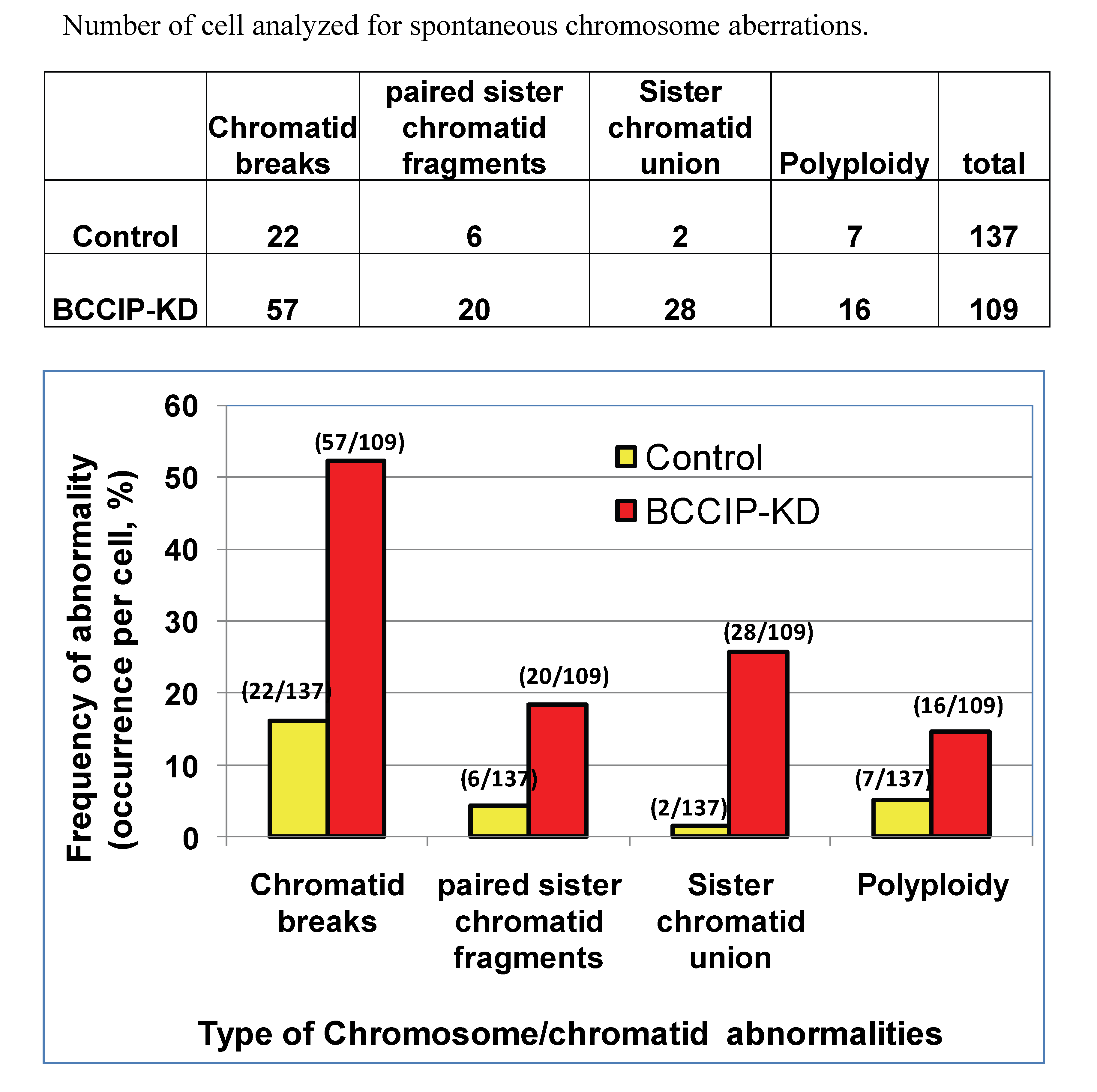

Supplement: Figure S6 — Spontaneous chromosome aberrations in BCCIP deficient MEFs. Shown are the frequencies of three types of chromatid abnormalities in 109 controls and 137 BCCIP-knockdown cells. (TIF) [file pgen.1002291.s006.tif]

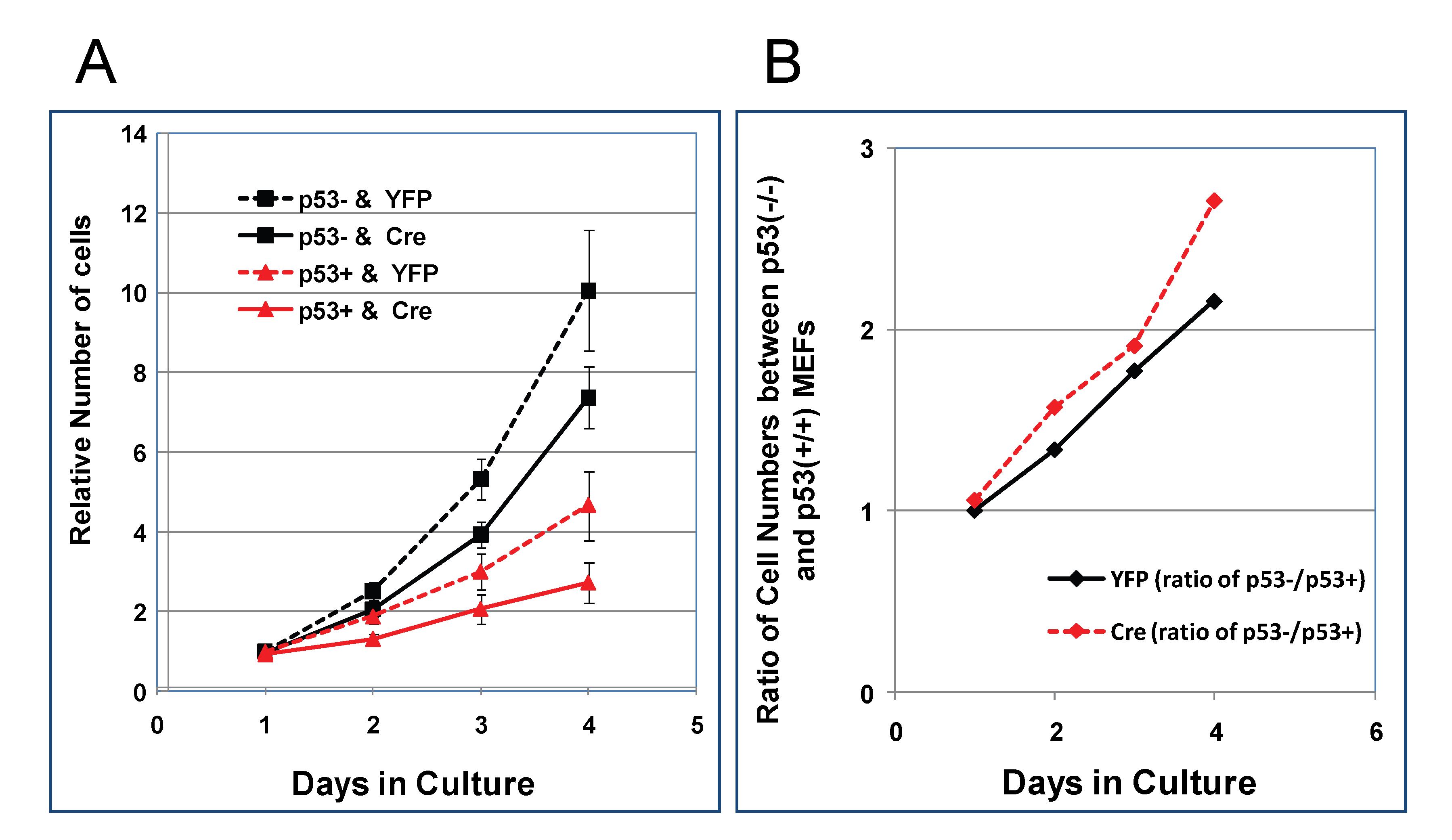

Supplement: Figure S7 — Partial rescue of growth retardation by p53 deletion in BCCIP deficient MEFs. Panel A shows the growth curves of BCCIP wild type (YFP) and knockdown (Cre) MEFs with wild type p53 (red lines) or null p53 (black lines). Data are average of 3 independent measurements, and each measurement had three replicas. As shown here, the p53 deletion resulted in a better growth in both control (YFP) and BCCIP knockdown (Cre) MEFs. However, with the p53 null background, the Cre-expressing (BCCIP knockdown) cells still grew slower compared to the controls. Panel B shows the ratios of cell numbers between p53 null and p53 wild type control (black line) and BCCIP deficient (red line) cells. As can be seen here, this ratio is higher for BCCIP deficient MEFs than that of the YFP control MEFs, suggesting a preferred stimulation of cell growth by null p53 in the BCCIP deficient cells than the control. (TIF) [file pgen.1002291.s007.tif]
