# Supplementary material for: Essential Roles of BCCIP in Mouse Embryonic Development and Structural Stability of Chromosomes
Source: PLoS Genet. 2011 Sep 22;7(9):e1002291. doi: 10.1371/journal.pgen.1002291 (PMC3178617; doi:10.1371/journal.pgen.1002291)
Supplement: Table S1 — Number of blastocysts analyzed. (DOC) [file pgen.1002291.s008.doc]

**Table S1, number of blastocysts analyzed**

|  | Control | BCCIP Knockdown |
| --- | --- | --- |
| No. of pregnant mice | 7 | 9 |
| Total blastocysts | 58 | 71 |
| Attached blastocysts | 28 | 28 |
| Detached blastocysts | 30 | 43 |
