# Supplementary material for: Essential Roles of BCCIP in Mouse Embryonic Development and Structural Stability of Chromosomes
Source: PLoS Genet. 2011 Sep 22;7(9):e1002291. doi: 10.1371/journal.pgen.1002291 (PMC3178617; doi:10.1371/journal.pgen.1002291)
Supplement: Table S2 — Number of viable newborns obtained from three different breeding strategies. (DOC) [file pgen.1002291.s009.doc]

Table S2, number of viable newborns obtained from three different breeding strategies

| (p53+/+;LoxPshBCCIP+/+) mated with (p53+/+; EIIaCre+/-): 15 litters | |  |
| --- | --- | --- |
| genotypes | No. of Vialble Newborns | expected ratio |
| p53+/+ LoxPshBCCIP+/- EIIaCre+/- | 8 | 8/16 |
| p53+/+ LoxPshBCCIP+/- EIIaCre-/- | 68 | 8/16 |
| Total | 76 |  |
|  |  |  |
| (p53+/- LoxPshBCCIP+/+) mated with (p53+/-; EIIaCre+/-): 11 litters | |  |
| genotypes | No. of Vialble Newborns | expected ratio |
| p53+/- LoxPshBCCIP+/- EIIaCre+/- | 9 | 4/16 |
| p53+/- LoxPshBCCIP+/- EIIaCre-/- | 33 | 4/16 |
| p53-/- LoxPshBCCIP+/- EIIaCre+/- | 0 | 2/16 |
| p53-/- LoxPshBCCIP+/- EIIaCre-/- | 17 | 2/16 |
| p53+/+ LoxPshBCCIP+/- EIIaCre+/- | 6 | 2/16 |
| p53+/+ LoxPshBCCIP+/- EIIaCre-/- | 10 | 2/16 |
| Total | 75 |  |
|  |  |  |
| (p53+/-; LoxPshBCCIP+/-) mated with (p53+/-; EIIaCre+/-): 19 litters | |  |
| genotypes | No. of Vialble Newborns | expected ratio |
| p53+/- LoxPshBCCIP+/- EIIaCre+/- | 2 | 2/16 |
| p53+/- LoxPshBCCIP+/- EIIaCre-/- | 39 | 2/16 |
| p53-/- LoxPshBCCIP+/- EIIaCre+/- | 0 | 1/16 |
| p53-/- LoxPshBCCIP+/- EIIaCre-/- | 7 | 1/16 |
| p53+/+ LoxPshBCCIP+/- EIIaCre+/- | 2 | 1/16 |
| p53+/+ LoxPshBCCIP+/- EIIaCre-/- | 7 | 1/16 |
| p53+/- LoxPshBCCIP-/- EIIaCre+/- | 32 | 2/16 |
| p53+/- LoxPshBCCIP-/- EIIaCre-/- | 31 | 2/16 |
| p53-/- LoxPshBCCIP-/- EIIaCre+/- | 11 | 1/16 |
| p53-/- LoxPshBCCIP-/- EIIaCre-/- | 9 | 1/16 |
| p53+/+ LoxPshBCCIP-/- EIIaCre+/- | 8 | 1/16 |
| p53+/+ LoxPshBCCIP-/- EIIaCre-/- | 6 | 1/16 |
| Total | 154 |  |
